# Knowledge, Attitudes and Practices toward Dengue Fever, Vector Control, and Vaccine Acceptance Among the General Population in Countries from Latin America and Asia Pacific: A Cross-Sectional Study (GEMKAP)

**DOI:** 10.3390/vaccines11030575

**Published:** 2023-03-02

**Authors:** Asrul Akmal Shafie, Edson Duarte Moreira, Alberta Di Pasquale, Dirk Demuth, Joanne Yoong Su Yin

**Affiliations:** 1 Discipline of Social and Administrative Pharmacy, School of Pharmaceutical Science, Universiti Sains Malaysia, George Town 11800, Malaysia; 2 Associação Obras Sociais Irmã Dulce Hospital Santo Antônio and Oswaldo Cruz Foundation, Brazilian Ministry of Health, Salvador 40420-000, Brazil; 3 Regional Medical Affairs, Growth and Emerging Markets, Takeda Pharmaceuticals International AG Singapore Branch, Singapore 018981, Singapore; 4 Evidence Generation and Publications, Growth and Emerging Markets, Takeda Pharmaceuticals International AG Singapore Branch, Singapore 018981, Singapore; 5 Research for Impact, Singapore 159964, Singapore

**Keywords:** dengue, vaccine, vector control, knowledge, attitude, practice (KAP), capability, opportunity, motivation, behavior (COM-B), population survey, Latin America, Asia Pacific

## Abstract

Dengue represents a major public health concern. With effective vaccines in development, it is important to identify motivational factors to maximize dengue vaccine uptake. A cross-sectional, quantitative, electronic survey was administered to a nationally representative adult population (*n* = 3800) in Argentina, Brazil, Colombia, Mexico, Indonesia, Malaysia, and Singapore. Willingness to vaccinate against dengue, and Knowledge, Attitudes, and Practices (KAP) toward dengue, vector control, prevention, and vaccination were determined. The Capability, Opportunity, Motivation for Behavior change (COM-B) framework was used to identify factors correlated with dengue vaccine(s) uptake. KAP scores (standardized, 0–100% scale) resulted in a low global score for Knowledge (48%) and Practice (44%), and a moderate score for Attitude (66%); scores were comparable across countries. Of all respondents, 53% had a high willingness (Score: 8–10/10) to vaccinate against dengue, which was higher (59%) in Latin America (Argentina, Brazil, Colombia, Mexico) than in Asia Pacific (40%) (Indonesia, Malaysia, Singapore). Key factors significantly (*p* < 0.05) associated with increased willingness to vaccinate included accessibility to the public (subsidies and incentives) and trust in the healthcare system and government. A common approach to dengue prevention across endemic countries––with some country-specific customization, including education, vaccination, and vector control (multi-pronged)––may reduce dengue burden and improve outcomes.

## 1. Introduction

Dengue is one of the most widespread vector-borne viral diseases and continues to spread rapidly [1]. It is transmitted by the bite of female mosquitoes of the species *Aedes aegypti* (primary vector) and *Aedes albopictus* (secondary vector) [2] and is associated with a high morbidity rate, causing death in up to 20% of those who contract severe dengue [3]. The number of dengue cases reported to the World Health Organization (WHO) has increased tenfold over the past two decades, rising from 505,430 cases in 2000 to over 2.4 million cases in 2010, and 5.2 million cases in 2019. Reported deaths between 2000 and 2015 increased from 960 to 4032 [4]. A recent Dengue Global Burden of Diseases assessment report looking at epidemiological trends over 30 years (1990–2019) showed that higher death rates occurred in children <5 years and in older adults >70 years, with a gradually decreasing trend in children and an increasing trend in older adults. [5]. Both the total number of cases and the number of reported deaths decreased during the COVID-19 pandemic in 2020 and 2021 [4]; however, several countries witnessed a resurgence of dengue in 2022 [6]. 

The increasing incidence and prevalence of dengue over the past two decades has been linked to demographic and social changes, including climate change, unprecedented population growth, rapid urbanization, increased migration of populations, and the collapse of public health infrastructure [1]. Approximately half of the global population currently lives in areas that are environmentally conducive to dengue transmission [7]. According to a report by the WHO, dengue fever is currently endemic in over 125 countries and affects more than 3.6 billion people living in endemic areas [3]. The Americas, South-East Asia, and the Western Pacific region are the most severely affected regions, with over 70% of the global burden of dengue disease borne by Asia [8]. Dengue has been highlighted as a global health priority by the WHO [3] and has resulted in significant economic and clinical burdens in endemic countries [1]. The global burden of dengue has been estimated to be between USD 8.9 billion [9] and USD 39.3 billion, including costs of dengue treatment and costs due to lost productivity [10].

Due to a lack of medications specifically targeted to treat dengue viral infection, the control of dengue fever is dependent primarily on vector control (reducing contact with vectors spreading dengue viruses), as well as appropriate disease management. Means of vector control in endemic or partially endemic countries include personal and household prevention methods, such as using mosquito nets and coils, draining stagnant water regularly, and using mosquito repellents (e.g., sprays and creams). Vector control also relies on community methods, such as mosquito fogging and the Wolbachia program. Wolbachia is a naturally occurring bacteria that reduces the ability of *Aedes* mosquitoes infected with it to transmit dengue or other vector-borne viruses. 

In addition to vector control, there is a need for further tools to address the burden of dengue [11]. For vaccine-preventable diseases such as dengue, vaccination can be an effective way of protecting the at-risk population. The first vaccine against dengue (“CYD-TDV”) was licensed in 2015. However, after 1–2 years, when new findings showed increased risks for individuals not previously exposed to dengue, regulatory authorities and the WHO were prompted to restrict vaccination to subjects previously exposed to dengue. In the Philippines, public and political outcry subsequently led to distrust of the dengue vaccine as well as other government-led health interventions. Vaccine Confidence Project^TM^ surveys conducted before and after the controversy showed a dramatic drop in willingness to vaccinate and confidence in the effectiveness of vaccines in the Philippines. Recently, another dengue vaccine [TAK-003] was licensed by the European Medicines Agency in 2022 and the local health authority in Indonesia, and other vaccines are in the late stages of development [11]. With new vaccines on the horizon and the critical lessons learnt from the CYD-TDV controversy in the Philippines, it is imperative to first understand the attitude and perspective of potential target populations in order to support implementation pathways that can maximize future vaccine uptake. 

Given the lack of population-level information on the willingness to vaccinate against dengue in many countries, the first objective of this study was to understand dengue perception and vaccine acceptance among the general population in the target countries. Secondly, the study aimed to identify key factors that might contribute to vaccine confidence, motivation to practice vector control, and potential uptake of future dengue vaccine(s). The study focused on selected countries in Latin America and Asia Pacific with dengue-endemic areas at a national or regional level. This included some hyperendemic regions, where there is a higher need for a vaccine to minimize the dengue burden and protect vulnerable populations.

### Approach

The Knowledge, Attitudes, and Practices (KAP) framework was used to evaluate individuals’ level of Knowledge of dengue disease and prevention; Attitudes toward the risk of contracting dengue and the effectiveness of vector control methods; and Attitudes and Practices toward dengue community vector control, personal prevention, and vaccination. Increasingly, KAP studies are recognized by vaccine technical advisory committees at the national and supranational levels (e.g., WHO, US Centers for Disease Control and Prevention (CDC)) as crucial in informing new vaccine recommendations and policies [12,13,14]. To enhance the understanding of KAP toward dengue fever and its prevention, the Capability, Opportunity, Motivation, Behavior (COM-B) model was used to structure the analysis. The COM-B model maps out the interaction between an individual’s Capability to perform a Behavior, the Opportunity to engage in that Behavior, and the Motivation that directs the occurrence of that Behavior at a given moment [15]. Studies have shown that interventions based on theoretical modelling of behavior are more effective than non-theoretical interventions [16,17,18]. For this study, the COM-B model was chosen because it provides not only a theoretical analysis of factors that could influence behavior change, but also evidence-based results that can be used to design appropriate interventions [19]. Moreover, the COM-B model has been adopted in a number of studies and has been used to improve understanding of vaccination practices and vaccine hesitancy [20,21].

Studies have been carried out to understand general vaccine confidence and barriers to vaccine uptake globally [22,23]; however, there is a lack of KAP studies focused on dengue vaccines among the general adult populations of endemic or partially endemic countries in Latin America and the Asia Pacific region. The majority of existing studies focused on specific populations or regions and, given the lack of consistency in measures taken across different countries, the factors impacting willingness to vaccinate against dengue are not fully understood at a country level [24,25,26,27,28,29,30,31]. Vaccine hesitancy is a complex phenomenon shaped by several factors, including vaccine availability, accessibility, cost, trust in the safety and efficacy of vaccines, perceived risk of the disease, confidence in governments and healthcare systems, and social pressures and norms. Understanding the determinants of vaccine hesitancy in a given population is crucial for the successful implementation of vaccination programs, as has been seen with COVID-19 vaccines [32,33].

## 2. Materials and Methods

### 2.1. Study Design

A cross-sectional, quantitative electronic survey was conducted among a nationally representative general adult population across seven countries in Latin America and Asia Pacific: Argentina, Brazil, Colombia, Mexico, Indonesia, Malaysia, and Singapore. The survey included 35 questions in the main survey, took approximately 30 min to complete, and was administered in local country languages. Data collection took place between September and October 2022.

### 2.2. Participants

Potential respondents were recruited through an existing, general purpose (i.e., not healthcare-specific) web-based consumer panel. Panelists joined voluntarily and were required to pass an industry-standard and panel-specific quality check to validate that respondents were not fraudulent. The majority of respondents accessed the survey through an email invitation from their panel provider and voluntarily completed the survey online. To ensure adequate representation of older age groups and those living in remote areas without reliable internet access, offline recruitment was also conducted by local partner fieldwork agencies. These respondents were invited to complete the survey at a partner center, in accordance with a Computer Assisted Web Interview (CAWI) methodology [34]. Participants who completed the full survey received an incentive in the form of points that could be exchanged for a small prize. 

Eligible participants included males and females of legal age (as per local country law) and up to 60 years of age who were able to provide consent to participate in the survey. The upper age limit for this survey was decided based on two main factors: first, the higher prevalence of dengue among children and young adults in endemic countries means that dengue prevention and management measures have a greater impact on younger age groups; second, restricting the survey to adults ≤60 years of age aimed to limit differences in the digital literacy of the survey participants, and thus, to reduce potential selection bias.

Individuals who had participated in other dengue-related surveys over the past three months and those who were not decision-makers for their health or not personally responsible for their health were excluded from the survey.

The study targeted between 400 and 600 respondents per country. A smaller sample size was planned for countries with smaller population sizes (i.e., Malaysia and Singapore). At the country level, this sample size would lead to no more than a 4.8% and 3.9% margin of error, respectively, for descriptive analysis. The target sample size was also in line with similar previously published studies [35]. A quota sampling procedure, with strata by gender, age, income, and region, was implemented to ensure that the sociodemographic composition of the study sample was representative of the adult population in each country with respect to gender, age, income, and region. Income levels were ranked based on each country’s socio-economic classifications and definitions of high, medium, and low income. Quotas for each stratum were pre-determined based on country census data and other publicly available data (e.g., United Nations Statistics Division, Central Intelligence Agency World Factbook, Department of Statistics Singapore) [36,37,38,39,40,41,42,43,44].

### 2.3. Electronic Survey Development

A new survey was developed for this study following the guidelines suggested by Tsang et al. [45]. The open survey collected data on Knowledge of dengue, Attitudes toward dengue, Practices of dengue vector control and mosquito bite prevention, Knowledge of and Attitudes toward dengue vaccination and vaccine roll-out, and trusted channels of data to disseminate health information. Responses were elicited as binary (true or false), Likert scale, multiple choice, and open-ended. The survey was developed in English (Supplementary File S1) and then translated into the native language of the selected countries. For each translation, a bilingual speaker initially performed a forward translation into the local language, and another bilingual speaker performed a backward translation. Two cognitive qualitative interviews were conducted in each of the target countries to refine, optimize, and validate the survey for comprehension, retrieval, decision-judgment, and response in the local languages. The survey incorporated adaptive questioning to conditionally display specific questions based on previous responses to reduce the number and complexity of the questions. Each of the 35 questions in the main survey was shown on a separate screen without the randomization of answer options. Respondents were required to complete a question before moving on to the next question to ensure the completeness of the survey responses. Respondents were not able to review or change their answers once a question had been answered. 

Data validation checks were programmed into the online survey to minimize entry errors by participants, and mandatory constraints and data quality assessments (Internet Protocol verification, identity validation, respondent digital fingerprinting, and engagement checks) were put in place. Each participant was allowed to answer the survey only once and any duplicates were removed; respondents were required to answer all questions. Following data collection, the collated data were reviewed and assessed using a data cleaning program, which identified straight-line answers on multiple choice questions, unusual patterns in the data, atypical time stamps, and inconsistent or nonsensical data in text-free questions.

### 2.4. Covariates and Outcomes

Sociodemographic variables including gender, age, household size, ethnicity, religion, region of residence, level of education, and household income were collected from all respondents. Other baseline characteristic variables collected through the survey included dengue experience (whether the respondent had previously contracted dengue), level of perceived risk (low, medium, high), history of dengue vaccination, COVID-19 vaccination, and history of influenza vaccination. All data used in the study were self-reported from the survey.

The study’s primary outcome was the respondents’ willingness to be vaccinated against dengue. Willingness to be vaccinated was measured using the Juster Scale, an eleven-point numerical scale ranging from 0 (no chance, almost no chance) to 10 (certain, almost certain), with each point associated with both a verbal and a numerical probability statement. A higher score indicated a higher willingness to be vaccinated, with a score of 8 to 10 considered a “high” willingness to vaccinate. 

The secondary outcome of the study was overall Knowledge, Attitudes, and Practices toward dengue infection and symptoms, dengue prevention methods, and dengue vaccines. Each survey question was assigned to a subcategory of KAP, and composite scores were derived for each subcategory (Supplementary Table S1). Overall K, A, and P composite scores were also derived and standardized to a scale of 0–100%. For K, A, and P scores within each country, 80–100% was considered a “high” score, 50–80% a “moderate” score, and 50% or below a “low” score. For Attitude scores, a higher score indicated a more positive Attitude.

The COM-B framework was used to identify explanatory variables. The three basic constructs of the COM-B framework—Capability, Opportunity, and Motivation—were derived from a specific criterion (Supplementary Table S2). Composite C, O, and M scores were used as explanatory variables to understand their relationship with willingness to be vaccinated.

### 2.5. Statistical Analysis

Sociodemographic and other baseline characteristic variables, as well as primary and secondary outcome variables, were reported descriptively using counts and percentages for categorical variables and means for continuous variables. Other survey data were also analyzed descriptively.

Bivariate comparison of the primary outcome (willingness to be vaccinated against dengue) across sociodemographic and other baseline subgroups (age, gender, household size, level of education, ethnicity, religion, income level, prior dengue experience, perceived risk of dengue, prior experience with dengue, COVID-19, or influenza vaccine, and perceived usefulness of vaccines in general) were performed using one-way analysis of variance (ANOVA) to understand the differences in willingness across these subgroups.

Subgroup analysis of the secondary outcome was also conducted to ascertain differences in KAP levels according to age group, sex, income, education level, endemic vs. non-endemic regions, ethnicity, religion, dengue experience, perceived level of risk from dengue, experience taking the COVID-19, influenza, or dengue vaccine, and overall vaccine acceptance. In this study, endemic regions were defined as regions that are moderately to highly endemic relative to the endemicity of the entire country. 

Multivariate regression analysis was deployed to identify and understand the key behavior change factors that may be associated with the potential uptake of a vaccine, using generalized linear models. The dependent variable in all regression models was an individual’s willingness to be vaccinated against dengue, as captured by the Juster scale (0–10). Six sets of regression were conducted, with each regression focusing on selected factors within the C, O, M categories of the COM-B framework (i.e., psychological capability, physical capability, physical opportunity, social opportunity, automatic motivation, and reflective motivation), respectively (Supplementary Table S2). These C, O, M factors were selected for inclusion in the models if they were individually correlated with willingness to be vaccinated at a univariate level. Sociodemographic and other baseline characteristics (age, gender, household size, level of education, ethnicity, religion, income level, prior dengue experience, prior experience with influenza vaccine, and perceived usefulness of vaccines in general) were determined a priori to be included as covariates in all sets of regressions. This was to account for potential differences in willingness to be vaccinated due to dissimilarities in these characteristics. Regression analysis was conducted at the global level (i.e., the entire sample) and at each individual country level. 

No data imputation was carried out for the analyses; where a response was missing for a variable included in the multivariate model, the participant was excluded from that analysis. For questions that included an option such as “do not know” or “do not wish to say,” such responses were considered complete but were omitted from some quantitative analyses. Statistical significance was determined at the 0.05 level. All data analyses were performed using R, version 4.2.1 [46].

### 2.6. Ethics and Data Confidentiality

Given the multi-country nature of the study and minimal risk to participants, the study was submitted to a central institutional review board, the Pearl Institutional Review Board (IN, US, Study Number: 22-VIST-101), for review and was granted exemption status. Prior to starting the survey, each respondent provided informed consent electronically. No personal identification information was collected, stored, or transferred during the survey. All data were handled in accordance with local data privacy laws in the study countries. Data were analyzed anonymously in aggregate and archived via a secured system with permission-based access.

## 3. Results

### 3.1. Survey Response Rates

**Figure 1:**
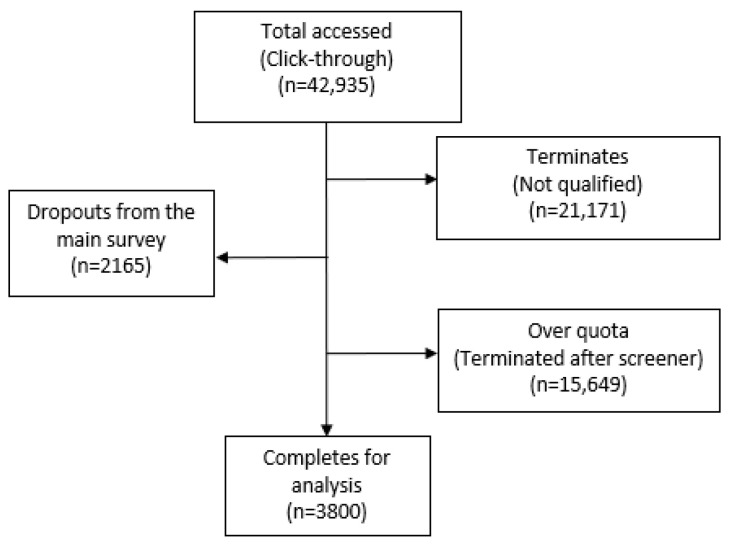
Flow chart of survey response rate.

Select questions were filtered to appear based on the respondent’s previous answer. The recruitment rate of respondents who accessed the screener questionnaire (*n* = 42,935), and whose answers were subsequently used for analysis, was 9% (*n* = 3800) (Figure 1). As quotas were set on age, income, and region, if such quotas were already met, even respondents who qualified to complete the questionnaire were not permitted to do so (i.e., “over quota”). Of the 3800 responses, 19 were collected via offline recruitment and used the CAWI methodology for data collection. All 3800 respondents answered the entire questionnaire, and no questions were left blank.

### 3.2. Sociodemographic Characteristics of Participants

A total sample size of 3800 respondents was analyzed for the global population. Most participants belonged to moderate-sized households of 3–4 members, with the global average household size being 3.5 (Table 1). Only a minority of respondents across all countries (14%) identified as having no religion, with the smallest minority found in Malaysia and Indonesia. Overall, respondents had a high level of education, with 50% or above having completed tertiary education in all countries apart from Argentina and Brazil, where 46% and 37% of respondents had completed tertiary education, respectively. Experience of having been previously infected with dengue varied between countries, with the lowest prior infection experience in Argentina (10%) and Singapore (15%), and the highest in Brazil (46%). Vaccination experience was higher in Latin America than in Asia Pacific. While almost all respondents were vaccinated against COVID-19 (94%), populations in Asia Pacific were less likely to be vaccinated against influenza compared with populations in Latin America (26% vs. 66%).

### 3.3. Knowledge, Attitudes, and Practices Toward Dengue 

**Figure 2:**
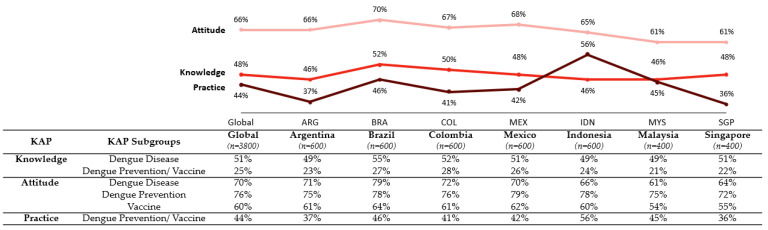
Knowledge, Attitudes, and Practices overall composite score and individual factor scores globally and by country.

KAP scores were relatively consistent across countries, with no major differences between Latin America and Asia Pacific (Figure 2). While overall Attitude scores were moderate (in the range of 60–70%), Knowledge and Practice scores were generally low to moderate (at or below 50%). KAP scores were consistently higher among populations with a positive opinion of vaccines in general, higher levels of perceived risk, prior dengue infection, and general vaccination experience (Supplementary Table S3). 

Brazil had the highest overall Knowledge score (52%), while Argentina, Indonesia, and Malaysia had the lowest (46%) (Figure 2). Education level was positively associated with overall dengue Knowledge, as was prior dengue infection, higher perceived risk of dengue, and positive opinion toward vaccines (Supplementary Table S3). Knowledge gaps concerned dengue disease, vector control prevention methods and their recommended frequency, and the availability of dengue vaccines. 

Brazil had the highest overall Attitude score among all countries (70%), while Singapore and Malaysia had the lowest (61%) (Figure 2). Attitude scores were relatively consistent within and across all sociodemographic subgroups, except for education level, which was positively associated with higher overall Attitude scores. While overall Attitude scores were generally higher than Knowledge scores, Attitudes toward vaccines were lower than Attitudes toward dengue disease and Attitudes toward dengue prevention methods (60% vs. 70% and 76%, respectively) (Figure 2). Despite the widespread perception of dengue as a moderate to severe disease, only 40% of respondents believed that dengue vaccination was more important than influenza vaccination.

Practice scores for dengue prevention measures (community and personal prevention measures against the vector) were relatively consistent across countries; however, confidence in prevention measures varied by country. Indonesia had a markedly higher Practice score (56%) than all other countries, while Singapore had the lowest score (36%) (Figure 2). Overall, Practice scores were higher for older age groups and for those with higher education levels, higher income, higher level of perceived risk, prior dengue infection, experience with vaccines, and a positive opinion toward vaccines (Supplementary Table S3).

### 3.4. Capability, Opportunity, and Motivation Toward Dengue, Dengue Prevention, and Vaccinations

**Figure 3:**
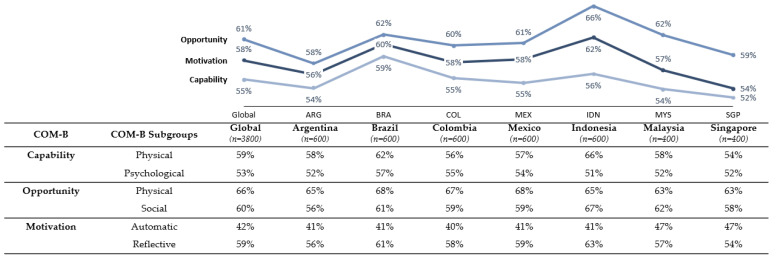
Capability, Opportunity, and Motivation overall composite score and individual factor scores globally and by country.

COM levels were generally moderate across all countries (in the range of 52–66%), with Capability and Motivation scores consistently lower than Opportunity scores (Figure 3). Argentina and Singapore had the lowest scores on all COM-B factors, while Indonesia had the highest Opportunity and Motivation scores. 

Overall, Capability was the lowest scoring factor compared with other COM-B factors. Moderate Capability scores highlighted knowledge gaps about dengue transmission, symptoms, and treatment methods. Physical and Psychological Capability scores (59% and 53% globally) were generally similar within a given country, apart from Indonesia, which showed markedly higher Physical Capability scores (66%) compared with Psychological Capability scores (51%). Overall, Capability levels did not vary significantly by subgroup, except for education level, which was associated with higher physical Capability. Capability levels varied more according to health experience than sociodemographic factors; respondents with prior dengue infection had a higher Capability score compared with those with no history of dengue, as did respondents with previous vaccine experience (Supplementary Table S4).

Populations across all countries perceived Physical Opportunities to participate in dengue prevention activities to be marginally higher than Social Opportunities (66% vs. 60%, globally). Perceptions of Physical and Social Opportunities differed mainly by age and whether respondents had children, with younger respondents and respondents with no children having a lower Opportunity score. Opportunity levels were higher for those with a more positive opinion toward vaccines in general, and those with a higher perceived risk of dengue.

Populations in Latin America had higher overall Motivation scores compared with Asia Pacific (59% vs. 42%), as well as a more positive outlook on the ability to manage dengue (55% vs. 39%). Respondents with higher education levels, prior dengue experience, and more positive vaccine opinion tended to have higher Motivation levels. Automatic Motivation levels were consistently lower across all countries compared with Reflective Motivation levels (42% vs. 59%, globally). While respondents across all countries generally recognized the importance of dengue prevention, there was a relative lack of incentives to receive a hypothetical dengue vaccine and deterrents for not vaccinating, even in countries where the dengue vaccine was available. 

Similar to KAP scores, COM scores were consistently higher among respondents with positive vaccine opinion, higher levels of perceived risk, prior dengue infection, and vaccination experience. Respondents with higher perception of dengue risk could identify and better leverage Social and Physical Opportunities compared with those with lower perceived risk, while respondents with a more positive opinion toward vaccines in general tended to be more knowledgeable about vaccine effectiveness and safety in general and more motivated to receive another vaccine.

### 3.5. Willingness to Vaccinate Against Dengue Disease

Dengue was generally perceived as a relatively severe (life-threatening) disease across both Latin America and Asia Pacific, with only 7% of all respondents considering dengue low risk (scores of 5 or below on a 10-point scale) (Supplementary Table S5). The standard deviation of the mean global score was 1.6, suggesting that most respondents agreed that dengue poses a moderate to high risk. Brazil, which has the highest percentage of its population previously infected with dengue, was also the country with the highest level of perceived risk (78% of respondents perceived dengue as severe, 8–10 on a 10-point scale), while Singapore had the lowest level of perceived risk (55% rating dengue as severe, 8–10 on a 10-point scale).

Perception of vaccines in general was markedly more positive in Latin America than in Asia Pacific, with Brazil registering the most positive vaccine opinion (with 79% scoring vaccines as very useful), and Singapore and Malaysia registering the least positive vaccine opinion (with 54% and 65%, respectively, scoring vaccines as very useful) (Supplementary Table S5).

Trends in willingness to vaccinate against dengue were similar to patterns seen for perceived dengue risk and perceived usefulness of vaccines. Globally, over 50% of respondents had a high willingness to vaccinate against dengue (Table 2). However, willingness to vaccinate against dengue was significantly higher in Latin America than in Asia Pacific; 60% of respondents in Latin America showed high willingness to vaccinate compared with 41% of respondents in Asia Pacific. Brazil reported the highest willingness to vaccinate, with 66% of respondents declaring that they were certain or almost certain to vaccinate compared with only 25% in Singapore. Overall, the study reflected high levels of willingness to vaccinate against dengue across all countries apart from Singapore and Malaysia, and to a lesser extent, Argentina (Table 2).

Globally, the most common reasons for willingness to be vaccinated were protection against dengue and protection of one’s health in general (18% and 15%, respectively) (Supplementary Table S6). Concerns over safety and efficacy were the most common reasons for not willing to be vaccinated against dengue, with 6% of respondents globally citing fear of side effects and 4% citing the belief that vaccines were not effective. The willingness to be vaccinated against dengue was similar across most sociodemographic subgroups, including gender, education level, and income. Respondents between 31 and 50 years of age had a higher willingness to be vaccinated compared with other age groups, as did respondents with children compared with respondents without children (Supplementary Table S6).

### 3.6. Behavioral Levers That Can Potentially Drive Vaccine Acceptance

Regression analyses identified key factors within the COM framework that were correlated with willingness to be vaccinated with a dengue vaccine (Supplementary Table S7). Factors that influenced willingness to vaccinate showed a high degree of consistency across countries, although some factors were specific to each country (Table 3 and Table 4). 

For the majority of countries, the perceived threat of dengue was positively correlated with willingness to be vaccinated. The belief that the vaccine should be made financially accessible to the population (*p*-value of <0.001 for all countries) as well as doctors’ recommendations of preventive vaccines were positively correlated with willingness to be vaccinated. Financial subsidies and incentives were generally important. Trust in the healthcare system and professionals (*p*-value of <0.001 for all countries), as well as the sponsorship of the community and/or government leaders also had a strong impact on willingness to be vaccinated (*p*-value of <0.001 for all countries; Table 3 and Table 4). 

For Colombia and Singapore, the number of personal and community mosquito prevention activities that respondents were currently practicing was positively associated with willingness to vaccinate (Colombia: *p*-value of 0.009, Singapore: *p*-value of 0.024). In Brazil and Argentina, the perception that the threat of dengue had been exaggerated by the media or government was negatively associated with willingness to vaccinate (Brazil: *p*-value of 0.018, Argentina: *p*-value of 0.401). Several other factors related to social opportunity also impacted vaccine willingness in Argentina, including recommendations from doctors or the government about upcoming vaccinations, and promotion of the importance of vaccines by social influencers, governments, and community leaders. In Mexico, Indonesia, and Singapore, community events promoting health were positively associated with willingness to vaccinate (Mexico: *p*-value of 0.036, Indonesia: *p*-value of <0.001, Singapore: *p*-value of 0.030). In all countries except Brazil and Mexico, incentives such as cash, points, or a gift were strongly positively associated with willingness to vaccinate.

### 3.7. Trusted Channels for Healthcare Information

Search engines and social media were the top two channels for the dissemination of health information across all countries (scores of 82% and 62%, globally) (Table 5, full list of channels and stakeholders included in Supplementary Table S8). Government or health agency websites or portals were more popular in Asia Pacific compared with Latin America (50% vs. 34%) (Table 6, full list of channels and stakeholders included in Supplementary Table S9). In Indonesia, social media and messaging platforms were especially popular (84%) compared with other countries. Overall, healthcare professionals were the most trusted stakeholders for health-related information, with 91% of all respondents citing doctors, 45% citing nurses and paramedics, and 36% citing pharmacists as trusted stakeholders. Trust in governments and scientific organizations varied widely across countries, with 66% of respondents in Singapore and 48% in Indonesia designating the government as a trusted stakeholder compared with only 20% in Argentina.

## 4. Discussion

To the best of the authors’ knowledge, this large multi-country study is the first of its kind to understand the Knowledge, Attitudes, and Practices toward dengue and dengue prevention, including willingness to vaccinate against dengue, among the general adult population in multiple endemic regions in Latin America and Asia Pacific. The study revealed several behavioral factors positively associated with willingness to vaccinate against dengue that could be considered when designing a vaccine implementation program. These include financial accessibility, social opportunity factors, disease educational interventions, and incentives. Interestingly, the KAP and COM scores were comparable across countries in Latin America and Asia Pacific, suggesting the possibility of a common dengue prevention approach that includes disease education, vaccination, and vector control. 

Study findings on KAP showed that knowledge levels on dengue infection, risk of transmission, and symptoms were moderate across all countries. This finding contrasts with previous KAP studies, which found knowledge gaps about dengue infection, particularly risk of transmission and symptoms, in Brazil and Argentina [46,47,48,49,50,51,52]; however, this difference may be related to the high education level of respondents in this survey. Similarly to prior KAP studies of specific regions or subpopulations within a single country, our study found that overall dengue knowledge was positively associated with higher education levels [53,54], as well as with higher perception of dengue risk and prior experience of dengue infection [55]. Although knowledge of dengue infection was moderate, the study showed low knowledge levels on the availability of dengue vaccines. This finding may suggest a lack of exposure and access to information about dengue vaccines, as has been shown with other adult vaccines [56,57,58]. Previous studies have suggested that clear recommendations by healthcare providers favoring vaccine uptake, as well as improved education programs by healthcare providers, can increase access to vaccine information in general [58]. Prior dengue KAP studies in Malaysia have also indicated the importance of educational campaigns in improving overall dengue knowledge, particularly among low-income or less-educated populations [59,60]. Less than half of the respondents in this study considered dengue vaccination to be more important than other optional vaccinations, despite the higher level of perception of the severity of dengue compared with diseases such as influenza. This may be due to a lack of knowledge about the availability of a dengue vaccine or to a belief that vector control measures are effective means of protecting against dengue. Perceptions of the effectiveness and safety of dengue prevention and vector control methods may have differed by country based on the different dengue strategies and education programs implemented in each country. 

The results of the multivariate regression analysis of covariates within the COM-B framework highlighted several individual, social, and systemic factors positively associated with willingness to vaccinate across all countries. Surprisingly, there was general agreement on the factors that influence willingness to be vaccinated against dengue across different countries in this study. This agreement may reflect the globalization of information about, and behaviors toward, infectious diseases and vaccination. Identification of common COM-B factors impacting vaccine willingness reveals the opportunity for a common dengue vaccine implementation approach; however, country nuances were also observed on specific factors, highlighting the need for customization by country based on local requirements. 

In relation to opportunity factors, the financial accessibility of vaccines had a significant impact on willingness to vaccinate. It was found in this study that people were more willing to get vaccinated if the vaccine was made available at no cost. As shown for other recommended vaccines [61,62,63], making vaccines accessible to the population through government or public sector subsidies may increase the acceptance of a dengue vaccine. Social opportunity factors with a high impact on willingness to vaccinate included the recommendations of doctors, governments, and community leaders. These stakeholders play a critical role in providing education, recommendations, and reminders about vaccination to people and communities to drive willingness and uptake of vaccines. Previous studies of other recommended vaccines have shown that the involvement of healthcare professionals in policy-making decisions regarding vaccine implementation, and the use of educational interventions in healthcare settings, can be effective in overcoming vaccine hesitancy [64,65]. Further investigation is required to understand the specific drivers and barriers for these stakeholders to advocate the importance of vaccination against dengue in each individual country context. Interestingly, in Colombia, the role of influencers in promoting the importance of vaccines was also significant, suggesting that a vaccine implementation program in Colombia may enlist the support of social influencers as well as doctors and governments. The analysis also showed a negative association between willingness to vaccinate and the belief that the risk of dengue has been exaggerated by the government or media. This association was seen especially in Argentina and Brazil, where a perception that dengue had been exaggerated or sensationalized by the government or media may have made people less sensitive to the severity of dengue, and therefore less willing to vaccinate against it. This finding highlights the need for improved methods of communicating the severity of dengue and the importance of dengue prevention to the public. Previous studies have shown that more effective health-related communication can be achieved by engaging communities in the development of messaging, focusing on unifying messages from different channels, addressing uncertainties promptly and with transparency, framing messages that aim to increase social responsibility and personal control, and diversifying modes of information dissemination. These strategies could potentially be useful in improving public awareness of dengue risks and prevention methods [66,67].

The analysis of motivation factors across countries revealed that incentives were powerful drivers of vaccination, whether these took the form of free vaccines, cash, or other incentives. Previous studies have shown that offering financial incentives to patients or clinicians has also been effective in improving rates of vaccination against influenza [68]. The current study showed that respondents who reported having trust in healthcare professionals and the healthcare system also had a higher willingness to vaccinate. This finding points to the importance of improving trust in healthcare systems, and to the need for further investigation of what factors drive trust and what governments and healthcare systems in individual countries can do to increase public confidence in these stakeholders. Finally, those who believed that vaccines were harmful and posed a high risk were less willing to get vaccinated. Overall, participants with a lower education level were more likely to believe that vaccines were harmful, underscoring the importance of education programs on the safety and effectiveness of vaccines, particularly programs targeting those with lower education levels. 

Studies aiming to understand populations’ KAP and COM toward vaccines are essential to tailoring vaccine implementation campaigns. Such studies should be conducted as an iterative process since external events such as the COVID-19 pandemic and the dengue CYD-TDV vaccination controversy in the Philippines can positively or negatively affect an individual’s willingness to vaccinate. By applying the COM-B framework, studies can enhance our understanding of how vaccine hesitancy and willingness to vaccinate evolve in a changing external environment and can shed light on how they might be addressed through public health interventions. 

When developing a dengue prevention roadmap, a multi-pronged approach should be adopted, encompassing vaccine implementation, disease education, and vector control. Combined control programs in Thailand have been shown to have the highest impact on disease burden compared with single interventions in terms of prevented dengue infections and lost disability-adjusted life-years [69]. There is a need for vaccination alongside vector control solutions, since although the risk of contracting dengue may be lower with vector control, the impact of dengue, if contracted, may be higher without vaccines. This is because in areas where vector control is practiced with success, the risk of dengue infection is lower and subsequently, population immunity is trending low [70,71]. Additionally, in areas where vector control is successful, as in the example of Singapore, willingness to vaccinate may be reduced since successful vector control may provide people with a false sense of security. The occurrence of a dengue outbreak in a more naïve population without exposure to the virus can cause a larger epidemic with more severe outcomes for those infected with dengue; therefore, vaccines are an important additional tool to increase population immunity. Previous studies have shown that vaccination and vector control programs can be conducted concurrently to maximize effectiveness in small or controlled environments [72]. Alongside vaccine implementation and vector control, disease and vaccination education is also an important element in increasing overall protection against dengue. The present study showed that participants were more inclined to participate in a vaccination program that also included an educational component. Education concerning population immunity (e.g., communicating the social benefits of population immunity and designing educational materials that visually demonstrate its effects) is also important in increasing understanding of disease prevention, and hence, willingness to get vaccinated, as shown in previous studies [73]. 

There are some limitations to this study. Firstly, although efforts have been taken to ensure the study sample is representative of the adult population in each country by setting quotas for gender, age, income, and region, potential bias may still exist in terms of other baseline characteristics such as education. Future KAP studies should consider setting a quota for level of education to ensure a more representative study sample. The survey aims and topics were deliberately not included in the survey invitation to reduce selection bias of respondents; however, this may not have been avoided fully among respondents who completed the survey, considering the high percentage of respondents vaccinated for dengue. Secondly, respondents self-reported their willingness to be vaccinated and it was not possible to validate the truthfulness of participants’ stated willingness to be vaccinated, or whether their stated willingness to be vaccinated would translate into getting vaccinated in reality. The self-reported nature of the survey may also be impacted by recall bias, as reflected in the relatively high reported rate of vaccinations against dengue and influenza. Moreover, this is a cross-sectional study, and it was not possible to establish a causal relationship between willingness to be vaccinated and KAP or COM scores. In cross-sectional studies, confounding variables may have been present; however, sociodemographic and other baseline characteristics were included in the multivariate regression to account for potential baseline differences and confounding effects when identifying the key behavior change factors that may be associated with willingness to be vaccinated. This large cross-region and cross-country study included respondents from both dengue-endemic and low-endemicity or non-endemic regions within a country. The study also considered current vector control and prevention tools within a community as well as a hypothetical willingness to vaccinate. Even with potential limitations, the quotas put in place allowed for a largely representative set of respondents and good generalizability of results.

## 5. Conclusions

Overall, there is a general level of agreement on the factors that influence willingness to be vaccinated against dengue across different countries in this study, with KAP and COM scores comparable across countries and regions. Dengue control approaches depend on multiple components: vector control, education, and vaccination. To effectively reduce the disease burden of dengue and protect at-risk populations, a multi-pronged program incorporating vector control methods and education as well as vaccination implementation appears to be the best approach. While country-specific nuances should be taken into account when designing individual vaccine implementation programs, the findings of this study reveal an opportunity for a common dengue vector control and vaccine implementation approach to be rolled out in Latin America and Asia Pacific. This approach should highlight the importance of harmonizing the implementation of various dengue control interventions. The KAP and COM-B framework is important in helping public health authorities, ministries of health and the environment, as well as other policymakers to further assess how to engage the population and identify behavior change factors to increase the acceptance of preventative measures against dengue, and dengue vaccination in particular. With the need to develop a multi-pronged approach to dengue prevention, this is an opportunity for environment and public health officials, policymakers, healthcare professionals, and community leaders to join efforts in addressing and shaping educational interventions on dengue prevention and control measures targeted at the general population, with the ultimate aim of reducing disease burden and improving health outcomes.

## Figures and Tables

**Table 1:** Sociodemographic characteristics of study respondents globally and by country.

Demographic	Sociodemographic	Global (*N* = 3800)	Argentina (*N* = 600)	Brazil (*N* = 600)	Colombia (*N* = 600)	Mexico (*N* = 600)	Indonesia (*N* = 600)	Malaysia (*N* = 400)	Singapore (*N* = 400)
Gender	Male	48%	49%	46%	49%	45%	51%	46%	47%
Age	18–30 years old	35%	36%	34%	38%	39%	31%	40%	23%
	31–40 years old	26%	25%	26%	25%	24%	28%	26%	26%
	41–50 years old	22%	22%	22%	21%	20%	24%	19%	26%
	51–60 years old	18%	17%	18%	16%	17%	18%	15%	26%
Household Size	Live alone	7%	15%	9%	5%	5%	4%	4%	8%
	1–2 members	19%	25%	31%	17%	15%	10%	13%	21%
	3–4 members	52%	44%	49%	54%	54%	60%	48%	53%
	5–6 members	19%	14%	10%	20%	22%	23%	26%	17%
	>6 members	4%	3%	1%	4%	5%	3%	9%	3%
Religion	Christianity	53%	68%	61%	83%	80%	14%	16%	27%
	Islam	18%	0%	0%	0%	0%	81%	43%	7%
	Buddhism or Taoism	8%	0%	0%	0%	0%	3%	32%	40%
	Hinduism	1%	0%	0%	0%	0%	0%	3%	4%
	Others	6%	8%	20%	4%	4%	2%	1%	2%
	No religion	14%	25%	19%	13%	16%	0%	5%	20%
Education Level	No formal education	0%	1%	1%	0%	0%	0%	0%	0%
	Primary education	2%	3%	6%	2%	1%	1%	0%	0%
	Secondary education	31%	46%	50%	27%	20%	31%	18%	14%
	Tertiary education	60%	46%	37%	63%	71%	64%	74%	73%
	Post-tertiary education	7%	4%	6%	8%	8%	4%	8%	13%
Level of Income	High	14%	10%	8%	5%	17%	15%	20%	30%
	Medium	43%	50%	39%	37%	40%	48%	40%	45%
	Low	43%	40%	53%	58%	43%	38%	40%	25%
Prior Dengue Infection	Yes	28%	10%	46%	31%	26%	38%	27%	15%
Vaccinated against COVID-19	Yes	94%	91%	94%	94%	94%	95%	97%	93%
Vaccinated against influenza	Yes	52%	53%	80%	57%	76%	21%	24%	37%

**Table 2:** Individual willingness to get vaccinated by a hypothetical dengue vaccine.

		Base	Mean	SD	High Willingness (Score: 10–8)		Medium Willingness (Score: 7–4)		Low Willingness (Score: 3–0)	
					n	%	n	%	n	%
Global	Global	3800	7.3	2.4	1996	53%	1513	40%	291	8%
Region	Latin America	2400	7.6	2.4	1428	60%	798	33%	174	7%
	Asia Pacific	1400	6.8	2.2	568	41%	715	51%	117	8%
Country	Argentina	600	7.2	2.7	319	53%	216	36%	65	11%
	Brazil	600	8.0	2.2	393	66%	179	30%	28	5%
	Colombia	600	7.4	2.5	333	56%	215	36%	52	9%
	Mexico	600	7.9	2.2	383	64%	188	31%	29	5%
	Indonesia	600	7.3	2.1	307	51%	259	43%	34	6%
	Malaysia	400	6.8	2.0	161	40%	211	53%	28	7%
	Singapore	400	6.0	2.2	100	25%	245	61%	55	14%
Gender	Male	1811	7.4	2.3	988	55%	696	38%	127	7%
	Pregnant women	109	7.4	2.2	60	55%	43	39%	6	6%
	Non-pregnant women	1880	7.2	2.5	948	50%	774	41%	158	8%
Age	Legal age–30 yo	1316	7.2	2.2	633	48%	593	45%	90	7%
	31–40 yo	977	7.4	2.4	529	54%	381	39%	67	7%
	41–50 yo	837	7.5	2.5	482	58%	288	34%	67	8%
	51–60 yo	670	7.2	2.6	352	53%	251	37%	67	10%
Endemic Region	Endemic	2318	7.3	2.3	1217	53%	942	41%	159	7%
	Low- or non-endemic	1482	7.3	2.5	779	53%	571	39%	132	9%
Wolbachia Region	Wolbachia cities	615	6.9	2.3	268	44%	249	48%	53	9%
	Non-Wolbachia cities	3185	7.4	2.4	1728	54%	1219	38%	238	7%
No. of Children	No children	1514	7.0	2.5	703	46%	662	44%	149	10%
	1–2 children	1912	7.5	2.3	1084	57%	712	37%	116	6%
	3–4 children	333	7.4	2.4	186	56%	124	37%	23	7%
	>4 children	41	7.4	2.2	23	56%	15	37%	3	7%
Education Level	No formal education	13	7.4	2.1	6	46%	6	46%	1	8%
	Primary education	84	7.4	2.6	46	55%	32	38%	6	7%
	Secondary education	1165	7.2	2.5	607	52%	450	39%	108	9%
	Tertiary education	2273	7.3	2.3	1196	53%	918	40%	159	7%
	Post-tertiary education	265	7.4	2.3	141	53%	107	40%	17	6%
Income	Low income	1651	7.3	2.5	871	53%	634	38%	146	9%
	Medium income	1618	7.3	2.3	848	52%	652	40%	118	7%
	High income	531	7.4	2.2	277	52%	227	43%	27	5%
Dengue Exp.	Previously contracted	1065	7.7	2.1	630	59%	393	37%	42	4%
	No dengue history	2735	7.2	2.5	1366	50%	1120	41%	249	9%
Level of Perceived Risk	Very high risk	1010	8.1	2.2	691	68%	279	28%	40	4%
	High risk	1200	7.3	2.1	614	51%	509	42%	77	6%
	Medium risk	1074	6.8	2.4	452	42%	517	48%	105	10%
	Low risk	265	6.8	2.6	117	44%	117	44%	31	12%
	Very low risk	185	6.8	2.9	88	48%	69	37%	28	15%
	No risk	66	6.8	3.2	34	52%	22	33%	10	15%
Vaccine Exp. (Influenza)	Vaccinated	1961	7.8	2.2	1219	62%	637	32%	105	5%
	Not vaccinated	1839	6.8	2.4	777	42%	876	48%	186	10%
Vaccine Exp. (COVID-19)	Vaccinated	3559	7.4	2.3	1941	55%	1397	39%	221	6%
	Not vaccinated	241	5.2	3.1	55	23%	116	48%	70	29%
Opinion Toward Vaccine	Very useful	2088	8.2	2.1	1480	71%	532	25%	76	4%
	Useful	1066	6.8	2.0	412	39%	569	53%	85	8%
	Somewhat useful	460	5.6	2.2	78	17%	313	68%	69	15%
	Slightly useful	127	5.1	2.4	20	16%	79	62%	28	22%
	Not useful	42	3.6	2.7	3	7%	19	45%	20	48%
	Not useful at all	17	2.2	3.9	3	18%	1	6%	13	76%

Yo: years old; exp.: experience.

**Table 3:** Capability, Opportunity, Motivation factors associated with willingness to vaccinate against dengue globally and by country in Latin America.

COM-B	Covariates ^1^	Global	Argentina	Brazil	Colombia	Mexico
		(*N* = 3800)	(*N* = 600)	(*N* = 600)	(*N* = 600)	(*N* = 600)
		COEFF	COEFF	COEFF	COEFF	COEFF
Capability (Physical)	I am afraid of needles	−0.006	0.004	−0.034	0.026	−0.010
	Number of dengue transmission activities currently practicing ^2^	**0.043**	0.044	−0.036	**0.098**	0.038
Capability (Psychological)	I do not think there is a vaccine that can prevent dengue	0.140	0.180	−0.320	0.375	−0.002
	I don’t know if there is a vaccine that can prevent dengue	−0.089	−0.184	**−0.839**	0.205	**0.511**
	There is a vaccine that can prevent dengue	Reference				
	Dengue is very severe	**0.162**	**0.147**	**0.146**	0.118	**0.272**
Opportunity (Physical)	The government has made it easy for people to get vaccinated by offering it at convenient locations	**0.065**	0.059	0.064	**0.156**	−0.043
	It is easy to schedule a vaccination appointment	**0.091**	0.041	**0.106**	0.057	**0.175**
	I believe that the vaccine should be made accessible to the public including myself	**0.262**	**0.328**	**0.253**	**0.261**	**0.223**
Opportunity (Social)	The threat of dengue is or has been exaggerated by the media	**−0.039**	−0.038	**−0.093**	−0.017	−0.040
	The threat of dengue is or has been exaggerated by the government	−0.015	**−0.079**	0.004	0.042	−0.024
	My doctor recommends vaccines for me and my family for several health conditions, as appropriate	**0.183**	**0.194**	**0.138**	**0.185**	**0.098**
	I receive reminders from doctors/the government about my upcoming vaccinations	**0.047**	**0.092**	0.051	0.050	0.007
	The government has broadcast education campaigns for people to get vaccinated	0.036	0.049	−0.016	**0.169**	−0.002
	My community/government leader(s) promote the importance of vaccines	−0.006	**−0.136**	**0.111**	−0.031	0.008
	My favorite influencer(s) promotes the importance of vaccines	0.012	**0.086**	0.014	**−0.088**	0.005
	My community organizes events promoting health	**0.047**	0.057	0.032	0.071	**0.067**
	My influencer(s) organizes events promoting good/improved health and well-being	**0.035**	0.043	−0.002	**0.085**	0.035
Motivation (Automatic)	If I have to pay for the vaccination, I will not do it	**−0.074**	**−0.094**	**−0.079**	−0.044	−0.050
	I will be more willing to get vaccinated if there are incentives (cash, points, or a gift)	**0.043**	**0.109**	0.015	**0.067**	0.006
Motivation (Reflective)	There is a high likelihood that people can contract dengue	**0.156**	0.139	0.161	0.033	0.186
	There is nothing we can do to treat dengue	0.017	0.019	0.044	0.030	0.005
	There is nothing we can do to prevent dengue	**0.044**	0.031	0.007	0.066	0.004
	We will all be completely powerless when it comes to dengue prevention	0.016	0.047	−0.013	−0.023	0.007
	We must accept it (dengue)	−0.019	−0.047	0.035	0.015	0.039
	I think vaccines are harmful	**−0.090**	**−0.147**	**−0.091**	−0.035	**−0.080**
	I trust the healthcare system and professionals in my country to deliver the vaccine and manage its side effects	**0.236**	**0.179**	**0.293**	**0.262**	**0.224**
	If the risk of contracting dengue is low, I may not get the dengue vaccine	**−0.095**	**−0.073**	**−0.083**	**−0.086**	**−0.086**
	My religious beliefs guide my health decisions	−0.013	0.058	−0.009	−0.022	−0.018
	The opinion of my community/government leader(s) is important to me	**0.071**	**0.112**	**0.085**	**0.074**	**0.065**
	The opinions of my influencer(s) are important to me	**0.067**	0.058	0.010	**0.084**	0.003

^1^ The covariates are statements that were asked on a Likert scale (0–10). A higher level of agreement with this statement is associated a with higher or lower willingness to get vaccinated. ^2^ The higher number of dengue transmission activities conducted is associated with a higher willingness to get vaccinated. Bold font indicates significant association at a *p*-value < 0.05. COEFF: Coefficient.

**Table 4:** Capability, Opportunity, Motivation factors associated with willingness to vaccinate against dengue globally and by country in Asia Pacific.

COM-B	Covariates ^1^	Global	Indonesia	Malaysia	Singapore
		(*N* = 3800)	(*N* = 600)	(*N* = 400)	(*N* = 400)
		COEFF	COEFF	COEFF	COEFF
Capability (Physical)	I am afraid of needles	−0.006	−0.026	−0.026	0.004
	Number of dengue transmission activities currently practicing ^2^	**0.043**	−0.033	0.015	**0.079**
Capability (Psychological)	I do not think there is a vaccine that can prevent dengue	0.140	0.246	0.150	0.160
	I don’t know if there is a vaccine that can prevent dengue	−0.089	−0.365	0.137	−0.200
	There is a vaccine that can prevent dengue	Reference			
	Dengue is very severe	**0.162**	**0.103**	**0.157**	0.187
Opportunity (Physical)	The government has made it easy for people to get vaccinated by offering it at convenient locations	**0.065**	**0.187**	**0.239**	−0.075
	It is easy to schedule a vaccination appointment	**0.091**	−0.040	0.036	**0.183**
	I believe that the vaccine should be made accessible to the public including myself	**0.262**	**0.295**	**0.200**	**0.248**
Opportunity (Social)	The threat of dengue is or has been exaggerated by the media	**−0.039**	−0.004	−0.020	−0.015
	The threat of dengue is or has been exaggerated by the government	−0.015	0.024	0.027	−0.029
	My doctor recommends vaccines for me and my family for several health conditions, as appropriate	**0.183**	**0.170**	0.100	**0.180**
	I receive reminders from doctors/the government about my upcoming vaccinations	**0.047**	**0.126**	−0.021	0.067
	The government has broadcast education campaigns for people to get vaccinated	0.036	**0.141**	**0.215**	−0.012
	My community/government leader(s) promote the importance of vaccines	−0.006	−0.045	−0.012	0.001
	My favorite influencer(s) promotes the importance of vaccines	0.012	−0.026	0.014	−0.017
	My community organizes events promoting health	**0.047**	**0.176**	0.033	**0.144**
	My influencer(s) organizes events promoting good/improved health and well-being	**0.035**	0.034	0.045	0.022
Motivation (Automatic)	If I have to pay for the vaccination, I will not do it	**−0.074**	**−0.107**	**−0.043**	**−0.090**
	I will be more willing to get vaccinated if there are incentives (cash, points, or a gift)	**0.043**	**0.087**	**0.093**	**0.127**
Motivation (Reflective)	There is a high likelihood that people can contract dengue	**0.156**	−0.027	−0.045	0.251
	There is nothing we can do to treat dengue	0.017	0.052	0.030	**0.088**
	There is nothing we can do to prevent dengue	**0.044**	0.031	0.071	0.043
	We will all be completely powerless when it comes to dengue prevention	0.016	0.008	−0.016	−0.054
	We must accept it (dengue)	−0.019	0.002	0.007	−0.046
	I think vaccines are harmful	**−0.090**	**−0.134**	**−0.099**	0.021
	I trust the healthcare system and professionals in my country to deliver the vaccine and manage its side effects	**0.236**	**0.165**	**0.237**	**0.348**
	If the risk of contracting dengue is low, I may not get the dengue vaccine	**−0.095**	−0.040	−0.072	**−0.232**
	My religious beliefs guide my health decisions	−0.013	0.021	0.008	−0.031
	The opinion of my community/government leader(s) is important to me	**0.071**	**0.192**	0.047	0.065
	The opinions of my influencer(s) are important to me	**0.067**	0.045	0.048	**0.122**

^1^ The covariates are statements that were asked on a Likert scale (0–11). Higher level of agreement with this statement is associated with a higher or lower willingness to get vaccinated. ^2^ The higher number of dengue transmission activities conducted is associated with a higher willingness to get vaccinated. Bold font indicates significant association at a *p*-value < 0.05. COEFF: Coefficient.

**Table 5:** Top 5 trusted channels for health-related information.

	Channels	Global (*N* = 3800)	Argentina (*N* = 600)	Brazil (*N* = 600)	Colombia (*N* = 600)	Mexico (*N* = 600)	Indonesia (*N* = 600)	Malaysia (*N* = 400)	Singapore (*N* = 400)
Channels for health-related information	Search engines	82%	81%	83%	81%	81%	88%	85%	77%
	Social media	62%	53%	57%	55%	60%	84%	75%	48%
	Television	44%	38%	54%	47%	41%	54%	36%	30%
	Government or health agency websites	40%	26%	42%	35%	35%	42%	54%	59%
	Websites specializing in health-related information	36%	28%	36%	36%	36%	40%	41%	38%

**Table 6:** Top 5 trusted stakeholders for health-related information.

	Stakeholders	Global (*N* = 3800)	Argentina (*N* = 600)	Brazil (*N* = 600)	Colombia (*N* = 600)	Mexico (*N* = 600)	Indonesia (*N* = 600)	Malaysia (*N* = 400)	Singapore (*N* = 400)
Trusted stakeholders for health-related information	Doctors	91%	91%	92%	93%	92%	94%	90%	81%
	Nurses or paramedics	45%	44%	49%	43%	47%	55%	44%	28%
	Pharmacists	36%	37%	43%	35%	26%	35%	52%	27%
	Government	34%	20%	32%	24%	25%	48%	39%	66%
	Scientific org.	34%	30%	40%	39%	35%	26%	34%	32%

## Data Availability

The data presented in this study are available on request from the corresponding author. The data are not publicly available.

## Supplementary Materials

The following supporting information can be downloaded at: https://www.mdpi.com/article/10.3390/vaccines11030575/s1, Supplementary Table S1: Definition and derivation of Knowledge, Attitude, and Practice subcategories of KAP framework; Supplementary Table S2: Definition and derivation of the Capability, Opportunity, and Motivation subcategories of COM-B framework; Supplementary Table S3: Overall level of Knowledge, Attitude, and Practice of respondents by subgroups; Supplementary Table S4: Overall level of Capability, Opportunity, and Motivation of respondents by subgroups; Supplementary Table S5: Perceived risk of dengue and usefulness of vaccines, generally; Supplementary Table S6: Rationale for willingness or unwillingness to receive a hypothetical dengue vaccine; Supplementary Table S7: Capability, Opportunity, and Motivation covariates used in the multivariate regression; Supplementary Table S8: Trust channels for health-related information; Supplementary Table S9: Trusted stakeholders for health-related information. A copy of the entire questionnaire is available as Supplementary File S1.
