# Supplementary material for: Knowledge, Attitudes and Practices toward Dengue Fever, Vector Control, and Vaccine Acceptance Among the General Population in Countries from Latin America and Asia Pacific: A Cross-Sectional Study (GEMKAP)
Source: Vaccines (Basel). 2023 Mar 2;11(3):575. doi: 10.3390/vaccines11030575 (PMC10058638; doi:10.3390/vaccines11030575)
Supplement: Supplementary file 1 [file vaccines-11-00575-s001.zip › vaccines-2189522-supplementary.pdf]

SUPPLEMENTARY MATERIALS

Supplementary Table S1 Definition and derivation of Knowledge, Attitude, and Practice sub-categories of KAP framework

	Sub-category	Definition	Calculation
**Knowledge**	Dengue infection and symptoms	Knowledge level on dengue transmission methods, serotypes of dengue, and diagnosis methods	Percentage of correct answers given by the respondent on questions regarding dengue disease and vector control
	Dengue vector control methods and vaccines	Knowledge level on dengue vector control prevention (methods and recommended frequency) and vaccines (vaccine safety and effectiveness; government, physician, or community leader endorsement; and dengue vaccine availability)	
**Attitude**	Dengue infection and symptoms	Attitudes regarding dengue disease (likelihood of contracting dengue, threat, Attitude toward government response) and severity of symptoms	Mean value of all Attitude-related questions on the Likert scale (0-10)
	Dengue prevention methods	Attitudes regarding dengue prevention (perceived effectiveness and safety of dengue vector control prevention measures, adherence to prevention measures at a societal level)	
	Vaccines	Attitudes regarding vaccination (factors that positively and negatively impacted decisions about vaccination, Attitudes toward the dengue vaccine at a personal and a societal level)	
**Practice**	Dengue prevention methods	Practice of dengue prevention focused on the number of and level of confidence in dengue prevention/ vector control measures performed by the individual	A function of the number of prevention methods listed by the respondent and the level of confidence ascribed to the prevention methods conducted

Supplementary Table S2 Definition and derivation of the Capability, Opportunity, and Motivation sub-categories of COM-B framework

	Sub-category	Definition	Calculation
**Capability**	Psychological	Individual's knowledge of and ability to comprehend information on the safety and effectiveness of dengue prevention methods including vaccine uptake	Percentage of correct and positive answers given by the respondent on questions regarding capability
	Physical	Individual's physical capacity, such as skill, dexterity, and strength, required to engage in activities to prevent dengue transmission and receive vaccines	
**Opportunity**	Physical	External physical factors that allow individuals to easily participate in dengue prevention activities and access vaccines, such as access to healthcare centers and information about dengue prevention and treatment	Percentage of positive answers given by the respondents on questions regarding opportunity
	Social	Social factors such as norms, expectations, conformity, and comparisons that influence an individual's behavior in undertaking dengue prevention activities and receiving vaccines	
**Motivation**	Automatic	Emotions and incentives, such as reinforcement through rewards or sense of fear, that influence an individual's behavior in undertaking dengue prevention activities and receiving vaccines	Percentage of positive answers given by the respondents on questions regarding motivation
	Reflective	Beliefs about capabilities or consequences and other conscious decision-making that motivates individuals to participate in dengue prevention activities and receive vaccines	

Supplementary Table S3 Overall level of Knowledge, Attitude, and Practice of respondents by subgroups

			n	Overall Knowledge	Overall Attitude	Overall Practice
**GLOBAL**			**3800**	**48%**	**66%**	**44%**
SOCIODEMOGRAPHICS	GENDER	Male	1811	47%	66%	43%
		Pregnant women	109	52%	62%	44%
		Non-pregnant women	1880	49%	66%	44%
	AGE	Legal age – 30 yo	1316	48%	64%	40%
		31 – 40 yo	977	49%	65%	44%
		41 – 50 yo	837	49%	67%	47%
		51 – 60 yo	670	47%	68%	46%
	ENDEMIC REGION[1]	Endemic	2117	49%	66%	45%
		Low or non-Endemic	1683	47%	66%	42%
	WOLBACHIA REGION	Wolbachia Cities	518	48%	63%	42%
		Non-Wolbachia Cities	3282	48%	66%	44%
	NO. OF CHILDREN	No children	1514	47%	65%	41%
		1 – 2 children	1912	49%	66%	45%
		3 – 4 children	333	50%	66%	46%
		>4 children	41	48%	63%	45%
	EDUCATION LEVEL	No formal education	13	46%	52%	27%
		Primary education	84	46%	64%	38%
		Secondary education	1165	47%	66%	41%
		Tertiary education	2273	49%	66%	45%
		Post-tertiary education	265	50%	66%	44%
	INCOME	Low income	1651	48%	66%	42%
		Medium income	1618	48%	66%	44%
		High income	531	49%	67%	46%
HEALTH EXPERIENCE	DENGUE EXP.	Previously contracted	1065	52%	66%	48%
		No dengue history	2735	47%	66%	42%
	LEVEL OF PERCEIVED RISK	Very high risk	1010	51%	69%	52%
		High risk	1200	49%	64%	44%
		Medium risk	1074	46%	65%	40%
		Low risk	265	47%	64%	37%
		Very low risk	185	44%	67%	34%
		No risk	66	42%	67%	26%
	VACCINE EXP. (Influenza)	Vaccinated	1961	51%	69%	46%
		Not vaccinated	1839	46%	63%	41%
	VACCINE EXP. (COVID-19)	Vaccinated	3559	48%	66%	44%
		Not vaccinated	241	45%	58%	37%
	OPINION TOWARDS VACCINE	Very useful	2088	50%	72%	50%
		Useful	1066	47%	60%	40%
		Somewhat useful	460	44%	55%	30%
		Slightly useful	127	45%	53%	29%
		Not useful	42	43%	50%	23%
		Not useful at all	17	45%	47%	30%

[1] Endemic regions are defined as regions which are moderately to highly endemic relative to the endemicity of the entire country.

Yo: year-old; exp.: experience

Supplementary Table S4 Overall level of Capability, Opportunity, and Motivation of respondents by subgroups

			n	Overall Capability	Overall Opportunity	Overall Motivation
	**GLOBAL**		**3800**	**55%**	**61%**	**58%**
SOCIODEMOGRAPHICS	**GENDER**	Male	1811	54%	61%	58%
		Pregnant women	109	57%	64%	57%
		Non-pregnant women	1880	56%	61%	58%
	**AGE**	Legal age – 30 yo	1316	53%	59%	56%
		31 – 40 yo	977	56%	62%	58%
		41 – 50 yo	837	57%	62%	59%
		51 – 60 yo	670	56%	62%	60%
	**ENDEMIC REGION[1]**	Endemic	2117	55%	62%	58%
		Low- or non-Endemic	1683	55%	60%	58%
	**WOLBACHIA REGION**	Wolbachia Cities	518	54%	61%	57%
		Non-Wolbachia Cities	3282	55%	61%	58%
	**NO. OF CHILDREN**	No children	1514	54%	58%	56%
		1 – 2 children	1912	56%	63%	59%
		3 – 4 children	333	57%	63%	59%
		>4 children	41	54%	63%	58%
	**EDUCATION LEVEL**	No formal education	13	46%	67%	49%
		Primary education	84	53%	59%	56%
		Secondary education	1165	54%	61%	57%
		Tertiary education	2273	55%	61%	59%
		Post-tertiary education	265	56%	61%	58%
	**INCOME**	Low income	1651	55%	61%	58%
		Medium income	1618	55%	61%	58%
		High income	531	56%	62%	59%
HEALTH EXPERIENCE	**DENGUE EXP.**	Previously contracted	1065	58%	63%	60%
		No dengue history	2735	54%	60%	57%
	**LEVEL OF PERCEIVED RISK**	Very high risk	1010	59%	67%	63%
		High risk	1200	55%	62%	58%
		Medium risk	1074	53%	58%	56%
		Low risk	265	52%	56%	54%
		Very low risk	185	51%	54%	54%
		No risk	66	50%	56%	53%
	**VACCINE EXP.** (Influenza)	Vaccinated	1961	57%	62%	60%
		Not vaccinated	1839	53%	60%	56%
	**VACCINE EXP.** (COVID-19)	Vaccinated	3559	55%	62%	59%
		Not vaccinated	241	51%	52%	51%
	**OPINION TOWARDS VACCINE**	Very useful	2088	58%	65%	63%
		Useful	1066	53%	60%	54%
		Somewhat useful	460	48%	52%	48%
		Slightly useful	127	50%	48%	46%
		Not useful	42	48%	39%	41%
		Not useful at all	17	52%	37%	41%

[1] Endemic regions are defined as regions which are moderately to highly endemic relative to the endemicity of the entire country.
Yo: year-old; exp.: experience

Supplementary Table S5 Perceived risk of dengue and usefulness of vaccines, generally

Variables	Levels of Perception	Global *(N=3800)*	Argentina *(N=600)*	Brazil *(N=600)*	Colombia *(N=600)*	Mexico *(N=600)*	Indonesia *(N=600)*	Malaysia *(N=400)*	Singapore *(N=400)*
**Level of perceived risk of dengue**	High perceived risk (Score: 8-10)	69%	71%	78%	72%	71%	66%	68%	55%
	Moderate perceived risk (Score: 6-7)	24%	24%	15%	21%	23%	25%	25%	38%
	Low perceived risk (Score: 0-5)	7%	6%	7%	7%	6%	10%	7%	7%
	Standard deviation	1.6	1.48	1.69	1.59	1.49	1.73	1.58	1.44
**Perception on the usefulness of vaccines** (general)	Very useful (Score: 8-10)	72%	70%	79%	77%	79%	74%	65%	54%
	Moderately useful (Score: 6-7)	17%	15%	14%	14%	14%	16%	21%	32%
	Not useful (Score: 0-5)	11%	15%	8%	9%	7%	10%	14%	14%
	Standard deviation	1.99	2.26	1.96	1.93	1.79	1.85	1.88	1.88

Supplementary Table S6 Rationale for willingness or no willingness to receive a hypothetical dengue vaccine

	**Rationale**	**Global** *(n=2605)*
**I will vaccinate**	Protect against dengue	18%
	Protect my health	15%
	Protect my family	8%
	Vaccine is important	7%
	Vaccine is safe	6%
	Increase immunity	5%
	Prevent epidemics	4%
	Reduce suffering from symptoms	4%
	Dengue is dangerous	3%
	Vaccine is effective	2%
	**Rationale**	**Global** *(n=1195)*
**I will NOT vaccinate**	Afraid of side effects	6%
	Vaccines are not effective	4%
	Research is not available	2%
	Vaccines are not required or necessary	2%
	No knowledge on vaccine	2%
	Afraid of vaccines	2%
	Vaccines are too new	2%
	It is/ will be expensive	1%
	There are a lot of vaccines	1%
	It is unsafe	1%

Supplementary Table S7 Capability, Opportunity, and Motivation covariates used in the multivariate regression

Score of 0= Strongly disagree, while 10= Strongly agree

Capability		Opportunity		Motivation	
**Physical**	**Psychological**	**Physical**	**Social**	**Automatic**	**Reflective**
**Q21.12**:<br>"I am afraid of needles"<br>Scored: 0-10	**Q10.2**:<br>"There is no vaccine yet that can prevent Dengue"<br>**Scored: Yes, no/false, don't know**<br>**Reference level: Yes**	**Q18.6**:<br>"The government has made it easy for people to get vaccinated by offering it at convenient locations"<br>Scored: 0-10	**Q9.3**:<br>"The threat of dengue is or has been exaggerated by the media"<br>Scored: 0-10	**Q22.3**:<br>"If I have to pay for the vaccination, I will not do it"<br>Scored: 0-10	**Q5.7**:<br>"Likelihood of people contracting dengue - Anyone"<br>Scored: 0-10
**Q11_COUNT** Which of the activities below are you currently practicing to prevent the transmission of dengue?<br>a) Spray insect repellent and/ or apply mosquito repellent patches<br>b) Participate in community mosquito fogging<br>c) Wear long-sleeved shirts and/ or long pants<br>d) Use wire mesh mosquito screens and/ or mosquito nets<br>e) Throw out any open bodies of water in plant containers, flower pots, tyres etc.<br>f) Perform periodic maintenance of water tanks<br>g) Tightly cover all water containers<br>h) Keep drain free of blockage<br>i) Place all garbage that can accumulate water into a closed bin<br>j) Add larvicide in water containers to kill mosquito larvae<br>k) None of the above	**Q4.** How severe is dengue<br><br>Scored: 0-10	**Q18.9**:<br>"It is easy to schedule a vaccination appointment"<br>Scored: 0-10	**Q9.4**:<br>"The threat of dengue is or has been exaggerated by the Government"<br>Scored: 0-10	**Q22.4**:<br>"I will be more willing to get vaccinated if there are incentives (cash, points, or a gift)"<br>Scored: 0-10	**Q9.1**:<br>"There is nothing we can do to treat dengue"<br>Scored: 0-10

**Q22.1**:<br>"I believe that the vaccine should be made accessible to the public including myself"<br>Scored: 0-10	**Q18.1**:<br>"My doctor recommends my family and I vaccines for several health conditions as appropriate"<br>Scored: 0-10		**Q9.2**:<br>"There is nothing we can do to prevent dengue"<br>Scored: 0-10
	**Q18.4**:<br>"I receive reminders from doctors/ the government about my upcoming vaccinations"<br>Scored: 0-10		**Q9.7**:<br>"We will all be completely powerless"<br>Scored: 0-10
	**Q18.5**:<br>"The government has broadcasted education campaigns for people to get vaccinated"<br>Scored: 0-10		**Q9.8**:<br>"We just have to accept it"<br>Scored: 0-10
	**Q18.7**:<br>"My community/ government leader(s) (e.g., governors, mayors, councilors etc.) promotes the importance of vaccines"<br>Scored: 0-10		**Q21.4**:<br>"I think vaccines are harmful"<br>Scored: 0-10
	**Q18.8**:<br>"My favorite influencer(s) (e.g., local and international celebrities, etc.) promotes the importance of vaccines"<br>Scored: 0-10		**Q22.2**:<br>"I trust the healthcare system and professionals in my country to deliver the vaccine and manage its side effects"<br>Scored: 0-10
	**Q30.2**:<br>"My community organizes events promoting health"<br>Scored: 0-10		**Q22.6**:<br>"If the risk of contracting dengue is low, I may not get the dengue vaccine"<br>Scored: 0-10

**Q31.2**:<br>"My influencer(s) organizes events promoting good/ improved health and well-being"<br>Scored: 0-10	**Q29.1** "My religious beliefs guide my health decisions"<br><br>Scored: 0-10 for each option
	**Q30.1**:<br>"The opinion of my community/ government leader(s) is important to me"<br>Scored: 0-10
	**Q31.1**:<br>"The opinions of my influencer(s) is important to me"<br>Scored: 0-10

Supplementary Table S8 Trusted channels for health-related information

	Channels	Global *(N=3800)*	Argentina *(N=600)*	Brazil *(N=600)*	Colombia *(N=600)*	Mexico *(N=600)*	Indonesia *(N=600)*	Malaysia *(N=400)*	Singapore *(N=400)*
**Trusted Channels for health-related information***	Search engines	82%	81%	83%	81%	81%	88%	85%	77%
	Social Media	62%	53%	57%	55%	60%	84%	75%	48%
	Television	44%	38%	54%	47%	41%	54%	36%	30%
	Government or health agency websites	40%	26%	42%	35%	35%	42%	54%	59%
	Websites specializing in health-related information	36%	28%	36%	36%	36%	40%	41%	38%
	Messaging platforms (e.g., WhatsApp, Telegram)	29%	20%	32%	22%	25%	50%	33%	23%
	Product and brand websites	27%	25%	20%	31%	26%	30%	31%	24%
	Newspapers, magazines	25%	19%	30%	27%	18%	24%	29%	32%
	Scientific journals (e.g., PubMed)	24%	19%	25%	27%	23%	29%	24%	19%
	Consumer reviews	21%	12%	18%	22%	17%	32%	27%	20%
	Radio	19%	19%	21%	28%	19%	13%	16%	13%
	Online forums, 'Question & Answer' services (e.g., Quora)	15%	8%	11%	12%	14%	29%	19%	16%
	Product and brand blogs/video blogs	13%	7%	11%	16%	12%	21%	17%	9%
	Patient advocacy websites	9%	6%	9%	11%	6%	12%	12%	5%
	Others	1%	2%	1%	1%	1%	0%	0%	0%

Supplementary Table S9 trusted stakeholders for health-related information

	Stakeholders	Global *(N=3800)*	Argentina *(N=600)*	Brazil *(N=600)*	Colombia *(N=600)*	Mexico *(N=600)*	Indonesia *(N=600)*	Malaysia *(N=400)*	Singapore *(N=400)*
**Trusted stakeholders for health-related information****	Doctors	91%	91%	92%	93%	92%	94%	90%	81%
	Nurses or Paramedics	45%	44%	49%	43%	47%	55%	44%	28%
	Pharmacists	36%	37%	43%	35%	26%	35%	52%	27%
	Government	34%	20%	32%	24%	25%	48%	39%	66%
	Scientific org.	34%	30%	40%	39%	35%	26%	34%	32%
	Family, friends and/ or colleagues	21%	18%	17%	19%	18%	33%	21%	22%
	Non-profit organizations	20%	18%	18%	20%	24%	15%	26%	22%
	Pharmaceutical companies	18%	11%	16%	18%	18%	27%	16%	16%
	Community leaders (e.g., village chiefs, community health leaders, etc.)	10%	5%	10%	9%	5%	19%	9%	13%
	Religious leaders	7%	3%	6%	5%	2%	18%	11%	4%
	Influencers (e.g., local and international celebrities, etc.)	5%	6%	5%	5%	3%	13%	4%	2%
	Others	1%	2%	1%	0%	1%	-	1%	1%

GEMKAP DENGUE STUDY SCREENER AND MAIN SURVEY

1.SCREENER

GENDER

S1. What is your gender?

❍ Male
❍ Female

AGE

S2. How old were you at your last birthday? Please enter your age in years.

❍ Younger than 17 years old
❍ 17-30 years old
❍ 31-40 years old
❍ 41-50 years old
❍ 51-60 years old
❍ Older than 60 years old

HOUSEHOLD SIZE

S3. How many people are living in your household including yourself?

By household we mean the number of people (including yourself) who live at the same address, who share cooking facilities and share a living room or sitting room or dining area.

❍ I live alone
❍ 1-2 members
❍ 3-4 members
❍ 5-6 members
❍ >6 members

S4. Are you a parent? If yes, how many children (below legal age) do you have?

❍ No children
❍ 1-2 children
❍ 3-4 children
❍ >4 children

S5. Are you currently pregnant?

If FEMALE is selected in S2;

❍ Yes
❍ No
❍ I don’t know

ETHNICITY

S6. Which of these best describes your ethnicity?

❍ Amerindian/Native American
❍ African

- ❍ Betawi
- ❍ Bumiputera
- ❍ Chinese
- ❍ Indian
- ❍ Javanese
- ❍ Madurese
- ❍ Malay
- ❍ Mestizo and White/European
- ❍ Others

RELIGION

S7. Which of these best describes your religion?

- ❍ Buddhism or Taoism
- ❍ Christianity
- ❍ Islam
- ❍ Hinduism
- ❍ Others
- ❍ No religion

REGION

S8. In which region do you live in?

- ❍ Argentina – Buenos Aires
- ❍ Argentina – Central
- ❍ Argentina – Cuyo
- ❍ Argentina – Patagonia
- ❍ Argentina – North-west
- ❍ Argentina – North-east
- ❍ Brazil – North
- ❍ Brazil – North-east
- ❍ Brazil – Central west
- ❍ Brazil – South-east
- ❍ Brazil – South
- ❍ Colombia – Amazon Region
- ❍ Colombia – Andean Region
- ❍ Colombia – Caribbean Region
- ❍ Colombia – Orinoquía Region
- ❍ Colombia – Pacific Region
- ❍ Mexico – North-west
- ❍ Mexico – North-east
- ❍ Mexico – Central north
- ❍ Mexico – Central south
- ❍ Mexico – West
- ❍ Mexico – East
- ❍ Mexico – South-west
- ❍ Mexico – South-east
- ❍ Indonesia – Sumatra
- ❍ Indonesia – Java

- ❍ Indonesia – Kalimantan
- ❍ Indonesia – Sulawesi
- ❍ Indonesia – Lesser Sunda Islands
- ❍ Indonesia – Maluku & Papua
- ❍ Malaysia – Northern
- ❍ Malaysia – East Coast
- ❍ Malaysia – Central
- ❍ Malaysia – Southern
- ❍ Malaysia – Eastern (Sarawak)
- ❍ Malaysia – Eastern (Sabah)
- ❍ Singapore – Central
- ❍ Singapore – East
- ❍ Singapore – North
- ❍ Singapore – North-east
- ❍ Singapore – West

S9. In which cities/ districts do you live in?

- ❍ Brazil – Belo Horizonte
- ❍ Brazil – Campo Grande
- ❍ Brazil – Petrolina
- ❍ Brazil – Niteroi
- ❍ Brazil – Rio de Janeiro
- ❍ Brazil – Others
- ❍ Colombia – Bello
- ❍ Colombia – Cali
- ❍ Colombia – Itagui
- ❍ Colombia – Medellin
- ❍ Colombia – Others
- ❍ Mexico – La Paz
- ❍ Mexico – Others
- ❍ Indonesia – Yogyakarta
- ❍ Indonesia – Others
- ❍ Malaysia – Kelantan
- ❍ Malaysia – Kuala Lumpur
- ❍ Malaysia – Putrajaya
- ❍ Malaysia – Penang
- ❍ Malaysia – Selangor
- ❍ Malaysia – Others
- ❍ Singapore – Bukit Batok
- ❍ Singapore – Choa Chu Kang
- ❍ Singapore – Tampines
- ❍ Singapore – Yishun
- ❍ Singapore – Others

EDUCATION

S10. What is your highest educational qualification?

- ❍ No formal education

- ❍ Primary education only, e.g. Primary school, elementary school
- ❍ Secondary education, e.g. Secondary school, middle school
- ❍ Tertiary education, e.g. College, bachelors, university-level, trade school
- ❍ Post tertiary education, e.g. Masters, PhD

DENGUE

S11. Have you contracted Dengue/Breakbone disease previously?

- ❍ Yes
- ❍ No

S12. Have you taken part in a Dengue/Breakbone disease survey in the past 3 months?

- ❍ Yes
- ❍ No
- ❍ I'm not sure

VACCINATION

S13. Are you a decision maker for your health?

- ❍ Yes, I am personally responsible for my health
- ❍ Yes, but I share the responsibility with someone else (e.g. Family, caregiver)
- ❍ No, someone else (e.g. Family, caregiver) is making the decision for me.

S14. Do you think vaccines are useful?

- ❍ YES, I have full confidence in most vaccines
- ❍ YES, I have some confidence in most vaccines
- ❍ YES, but I am hesitant about vaccines
- ❍ I have no opinions about vaccines
- ❍ NO, but I willing to consider vaccines
- ❍ NO, I don't believe vaccines are useful
- ❍ NO, I am strongly against vaccines, regardless of government approval

S15. Out of the optional vaccines below which vaccinations have you taken? Select all that apply.

- ❑ Dengue
- ❑ COVID-19
- ❑ Cholera
- ❑ Hepatitis A
- ❑ Hepatitis B
- ❑ HPV (Human Papillomavirus)
- ❑ Influenza
- ❑ Japanese Encephalitis
- ❑ Measles, Mumps and Rubella
- ❑ Meningitis (Meningococcal Vaccine)
- ❑ Rabies
- ❑ Tdap (Tetanus, Diphtheria and Pertussis)
- ❑ Tickborne Encephalitis
- ❑ Typhoid
- ❑ Varicella (Chicken Pox, Shingles Vaccine)
- ❑ Yellow Fever

❑ None of the above

INCOME

S16. And finally, which range best describes your MONTHLY HOUSEHOLD INCOME
Please consider ALL sources of revenue (including personal income, money sent from family overseas, revenue from investments, etc.)

- ❍ Argentina (ARS) - Low (<30,000)
- ❍ Argentina (ARS) - Mid (30,000 – 79,999)
- ❍ Argentina (ARS) - High (≥80,000)
- ❍ Brazil (BRL) - Low (<2000)
- ❍ Brazil (BRL) - Mid (2000 – 6999)
- ❍ Brazil (BRL) - High (≥7000)
- ❍ Colombia (COP) - Low (<900,000,000)
- ❍ Colombia (COP) - Mid (900,000 – 2699,999)
- ❍ Colombia (COP) - High (≥2700,000)
- ❍ Mexico (MXP) - Low (<9000)
- ❍ Mexico (MXP) - Mid (9000 – 39,999)
- ❍ Mexico (MXP) - High (≥40,000)
- ❍ Indonesia (IDR) – Low (<1500,000)
- ❍ Indonesia (IDR) – Mid (1500,000 – 4999,999)
- ❍ Indonesia (IDR) – High (≥5000,000)
- ❍ Malaysia (MYR) – Low (<5000)
- ❍ Malaysia (MYR) – Mid (5000 – 10,999)
- ❍ Malaysia (MYR) – Mid (11,000 – 10,999)
- ❍ Malaysia (MYR) – Mid (≥11,000)
- ❍ Singapore (SGD) – Low (<3000)
- ❍ Singapore (SGD) – Mid (3000 – 12,499)
- ❍ Singapore (SGD) – High (≥12,500)

2.MAIN STUDY SURVEY

DENGUE KNOWLEDGE

Q1. Please select true or false for the following statements.

	True	False
(a) Dengue is transmitted to a person via Aedes mosquitoes	❍	❍
(b) Aedes mosquitoes are more likely to bite in the evenings or at night	❍	❍
(c) Mosquitoes reproduce OR multiply in stagnant water	❍	❍
(d) Mosquitoes reproduce OR multiply in clear water	❍	❍
(e) Mosquitoes are more likely to bite when the weather is hot	❍	❍
(f) Mosquitoes are more likely to bite in humid weather. (Humidity refers to the amount of water vapor in the air. The higher the humidity, the higher the moisture in the air and the wetter it feels outside.)	❍	❍

Q2. Please select true or false for the following statements.

	True	False
(a) I live in an area where dengue is very common	❍	❍
(b) There are 4 different virus types of dengue	❍	❍
(c) You can only catch dengue once in your lifetime	❍	❍
(d) You can only catch dengue once in your lifetime	❍	❍
(e) I may be infected by 1 or more virus types of dengue at different points of time	❍	❍
(f) I cannot be infected by 2 or more virus types of dengue at the same time	❍	❍
(g) People can die from dengue and its related complications (e.g. fever, cough etc.)	❍	❍
(h) It is always possible to tell when someone has dengue by looking at them	❍	❍
	❍	❍

Q3. Have you or anyone else you know contracted dengue two times or more?

❍ Yes, I have contracted dengue at least two times or more
❍ Yes, I know someone who has contracted dengue at least two times or more
❍ No, I do not know anyone who has contracted dengue at least two times or more

Q4. How severe is dengue as a disease? Please rate each statement on a scale from 0 to 10, with 0 being 'not severe at all' and 10 being 'very severe'

	(0)	(1)	(2)	(3)	(4)	(5)	(6)	(7)	(8)	(9)	(10)	
Not severe at all												Very severe

Q5. Thinking about yourself and your family, community and neighborhood, how likely is it that the following people will contract dengue?
Please rate each statement on a scale from 0 to 10, with 0 being 'very unlikely' and 10 being 'very likely'.

		(0)	(1)	(2)	(3)	(4)	(5)	(6)	(7)	(8)	(9)	(10)	
a) Yourself	Very unlikely												Very likely

		(0)	(1)	(2)	(3)	(4)	(5)	(6)	(7)	(8)	(9)	(10)	
b) Family	Very unlikely												Very likely
c) Older residents who live in the neighborhood, aged 65 years old	Very unlikely												Very likely
d) People with chronic or long-term health conditions	Very unlikely												Very likely
e) Infants, children and adolescents of up to 18 years old	Very unlikely												Very likely
f) Adult residents, aged 18 - 65 years	Very unlikely												Very likely
g) Anyone	Very unlikely												Very likely

Q6. How is dengue diagnosed? Please select all that apply.

- ❑ Self-testing blood kit performed at home
- ❑ Self-testing nasal swab kit performed at home
- ❑ Blood test at the clinic or hospital
- ❑ Medical scans
- ❑ Signs/symptoms of the disease e.g. sore throat, cough, fever etc.
- ❑ Previous medical and travel history
- ❑ I do not know

Q7. Which of the following can often be signs of dengue? Please select all that apply

- ❑ Fever
- ❑ Headache
- ❑ Runny nose
- ❑ Flushing (face and/or body becomes red)
- ❑ Body aches/joint and muscle pain
- ❑ Swollen glands and lymph nodes
- ❑ Body rashes
- ❑ Body chills
- ❑ Hair loss (alopecia)
- ❑ Feeling very tired and sleepy
- ❑ Difficulty breathing
- ❑ Nausea and vomiting
- ❑ Loss of appetite
- ❑ Abdominal/ belly / stomach pain
- ❑ Pain behind the eyes
- ❑ Diarrhoea
- ❑ Bleeding from nose or gums
- ❑ All of the above

Q8. If someone were to contract dengue, what might the consequences be? Please select all that apply.

- ❑ Absenteeism from school/work
- ❑ Potential clinic visits
- ❑ Potential hospitalization
- ❑ Additional unexpected treatment costs from stay at hospital e.g. blood tests
- ❑ Additional costs due to caregiver requirements (incl. absence from work)
- ❑ Worsening of chronic and long-term health conditions
- ❑ Increased risk of contracting dengue again
- ❑ Increasing severity of dengue should it be contracted again
- ❑ Poorer quality of life
- ❑ All of the above

Q9. How much do you agree with each of the following statements?
Please rate each statement on a scale from 0 to 10, with 0 being strongly disagree' and 10 being 'strongly agree'.

		(0)	(1)	(2)	(3)	(4)	(5)	(6)	(7)	(8)	(9)	(10)	
a) There is nothing we can do to treat dengue	Strongly disagree												Strongly agree
b) There is nothing we can do to prevent dengue	Strongly disagree												Strongly agree
c) The threat of dengue is or has been exaggerated by the media	Strongly disagree												Strongly agree
d) The threat of dengue is or has been exaggerated by the government	Strongly disagree												Strongly agree
e) The government is responding appropriately to combat dengue	Strongly disagree												Strongly agree
f) The government is well prepared to combat dengue	Strongly disagree												Strongly agree
g) We will all be completely powerless	Strongly disagree												Strongly agree
h) We just have to accept it	Strongly disagree												Strongly agree

DENGUE PREVENTION

Q10. Based on your current knowledge about dengue, do you think the following statements are true or false?

- ❍ There are specific medicines that can cure dengue
- ❍ There is no vaccine yet that can prevent dengue

- ❍ There is a vaccine that can prevent dengue but it is not yet registered in my country

Q11. Which of the activities below are you currently practicing to prevent the transmission of dengue? Please select all that apply.

- ❑ Spray insect repellent and/ or apply mosquito repellent patches
- ❑ Participate in community mosquito fogging
- ❑ Wear long-sleeved shirts and/ or long pants
- ❑ Use wire mesh mosquito screens and/ or mosquito nets
- ❑ Throw out any open bodies of water in plant containers, flower pots, tyres etc.
- ❑ Perform periodic maintenance of water tanks
- ❑ Tightly cover all water containers
- ❑ Keep drain free of blockage
- ❑ Place all garbage that can accumulate water into a closed bin
- ❑ Add larvicide in water containers to kill mosquito larvae
- ❑ None of the above

Q12. How often do you complete these dengue prevention activities? Please select the answer closest to what you do.

- ❑ Always/ nearly all the time
- ❑ Every few hours/ at least twice a day
- ❑ Once a day
- ❑ Once every other day
- ❑ Once a week
- ❑ Once every two weeks
- ❑ Once a month
- ❑ Less than once a month
- ❑ As and when needed
- ❑ None of the above

Q13. How confident are you in completing these dengue prevention activities successfully? Please rate each statement on a scale from 0 to 10, with 0 being 'not confident at all' and 10 being 'very confident'.

		(0)	(1)	(2)	(3)	(4)	(5)	(6)	(7)	(8)	(9)	(10)	
a) Use wire mesh screens, mosquito nets and/or mosquito coils	Not confident at all												Very confident
b) Drain water from pots and cover all water containers	Not confident at all												Very confident
c) Community mosquito fogging	Not confident at all												Very confident
d) Spraying mosquito repellent and using larvicide to kill mosquito larvae	Not confident at all												Very confident

			(0)	(1)	(2)	(3)	(4)	(5)	(6)	(7)	(8)	(9)	(10)	
e)	Wolbachia program (WIAM)	Not confident at all												Very confident
f)	Dengue vaccination	Not confident at all												Very confident

Q14. How <u>effective</u> do you think each of the dengue prevention methods is for your personal health? Please rate each statement on a scale from 0 to 10, with 0 being 'not confident at all' and 10 being 'very confident'.

			(0)	(1)	(2)	(3)	(4)	(5)	(6)	(7)	(8)	(9)	(10)	
a)	Use wire mesh screens, mosquito nets and/or mosquito coils	Not safe at all												Very safe
b)	Drain water from pots and cover all water containers	Not safe at all												Very safe
c)	Community mosquito fogging	Not safe at all												Very safe
d)	Spraying mosquito repellent and using larvicide to kill mosquito larvae	Not safe at all												Very safe
e)	Wolbachia program (WIAM)	Not safe at all												Very safe
f)	Dengue vaccination	Not safe at all												Very safe

Q15. How <u>safe</u> do you think each of each of the following dengue prevention methods is for your personal health?
Please rate each statement on a scale from 0 to 10, with 0 being 'not safe at all' and 10 being 'very safe'.

			(0)	(1)	(2)	(3)	(4)	(5)	(6)	(7)	(8)	(9)	(10)	
g)	Use wire mesh screens, mosquito nets and/or mosquito coils	Not safe at all												Very safe
h)	Drain water from pots and cover all water containers	Not safe at all												Very safe

		(0)	(1)	(2)	(3)	(4)	(5)	(6)	(7)	(8)	(9)	(10)	
i) Community mosquito fogging	Not safe at all												Very safe
j) Spraying mosquito repellent and using larvicide to kill mosquito larvae	Not safe at all												Very safe
k) Wolbachia program (WIAM)	Not safe at all												Very safe
l) Dengue vaccination	Not safe at all												Very safe

Q16. How likely are you, your community/ neighborhood/ local council leader(s) or your government leader(s) (e.g. governors, mayors, councilors, etc.) to do the following dengue prevention activities in the next 6 months?
Please rate each statement on a scale from 0 to 10, with 0 being 'very unlikely' and 10 being 'very likely'.

		(0)	(1)	(2)	(3)	(4)	(5)	(6)	(7)	(8)	(9)	(10)	
a) Use wire mesh screens, mosquito nets and/or mosquito coils	Very unlikely												Very likely
b) Drain water from pots and cover all water containers	Very unlikely												Very likely
c) Community mosquito fogging	Very unlikely												Very likely
d) Spraying mosquito repellent and using larvicide to kill mosquito larvae	Very unlikely												Very likely
e) Wolbachia program (WIAM)	Very unlikely												Very likely
f) Dengue vaccination	Very unlikely												Very likely

DENGUE PREVENTION

Q17. How much do you agree with the statements below on vaccines as a preventative activity in general? Please rate each statement on a scale from 0 to 10, with 0 being 'strongly disagree' and 10 being 'strongly agree'.

			(0)	(1)	(2)	(3)	(4)	(5)	(6)	(7)	(8)	(9)	(10)	
a)	Vaccination is important for the prevention of certain diseases	Strongly disagree												Strongly agree
b)	All vaccines registered with the Ministry of Health are medically safe	Strongly disagree												Strongly agree
c)	I trust doctors to recommend safe and effective vaccinations	Strongly disagree												Strongly agree
d)	I trust pharmaceutical companies to produce safe and effective vaccinations	Strongly disagree												Strongly agree
e)	Vaccinations are only for children, the elderly and the vulnerable	Strongly disagree												Strongly agree
f)	I am pro-vaccination	Strongly disagree												Strongly agree
g)	I am more pro-vaccination after the COVID-19 pandemic	Strongly disagree												Strongly agree

Q18. Based on your past vaccination experience (in general), how much do you agree with the statements below?
Please rate each statement on a scale from 0 to 10, with 0 being 'strongly disagree' and 10 being 'strongly agree'.

			(0)	(1)	(2)	(3)	(4)	(5)	(6)	(7)	(8)	(9)	(10)	
a)	My doctor recommends my family and I vaccines for several health conditions as appropriate.	Strongly disagree												Strongly agree
b)	I make sure that my children's vaccination schedule is followed	Strongly disagree												Strongly agree
c)	I proactively ask my family's or children's doctor about vaccinations	Strongly disagree												Strongly agree
d)	I receive reminders from doctors/ the government about my upcoming vaccinations	Strongly disagree												Strongly agree

			(0)	(1)	(2)	(3)	(4)	(5)	(6)	(7)	(8)	(9)	(10)	
e)	The government has broadcasted education campaigns for people to get vaccinated	Strongly disagree												Strongly agree
f)	The government has made it easy for people to get vaccinated by offering it at convenient locations	Strongly disagree												Strongly agree
g)	My community/ government leader(s) (e.g. governors, mayors, councilors etc.) promotes the importance of vaccines	Strongly disagree												Strongly agree
h)	My favorite influencer(s) (e.g. local and international celebrities, etc.) promotes the importance of vaccines	Strongly disagree												Strongly agree
i)	It is easy to schedule a vaccination appointment	Strongly disagree												Strongly agree

Q19. Based on your past vaccination experience, please select all the statements that apply to your decision to take the influenza vaccine.

- ❑ The vaccination was free, discounted or the cost was claimed back from the government or insurance company
- ❑ I received an incentive (cash, points, or a present) to take the vaccine
- ❑ There was sufficient scientific evidence on the vaccine's safety and effectiveness
- ❑ I was aware of the consequences if I did not vaccinate
- ❑ I was not afraid of the vaccine's side effects
- ❑ The vaccine's benefits outweigh the side effects
- ❑ I felt that I was at high risk of contracting influenza
- ❑ It contributed to herd immunity (where a large part of the population is immune to influenza, reducing the spread of the disease in the general population)
- ❑ It was recommended by the government
- ❑ It was recommended by my doctor
- ❑ It is easy to get a vaccination appointment
- ❑ I heard about a friend/ family who had developed severe symptoms from influenza
- ❑ I wanted to protect my friends and family from contracting influenza
- ❑ My friends and family encouraged me to receive the vaccine
- ❑ I was given the vaccine during a routine visit to see a doctor
- ❑ **Others: ___**

Q20. Please select all the statements that apply to you not receiving the influenza vaccine.

- ❑ The vaccination was not free/ discounted/ claimed from the government or insurance company
- ❑ The vaccination was not affordable
- ❑ There was no incentive (cash, points, or a gift) to take the vaccine
- ❑ There was not sufficient scientific evidence on the vaccine's safety and how well it protects against influenza
- ❑ There are no consequences if I did not vaccinate
- ❑ I think that the vaccine is not safe
- ❑ I was afraid of the side effects that I might experience from the vaccine
- ❑ I experienced a side effect(s) from the vaccine and am reluctant to receive further vaccination
- ❑ The vaccines' side effects outweighed its benefits
- ❑ I do not think that I was at risk of contracting influenza
- ❑ It does not contribute to herd immunity (where a large part of the population is immune to influenza, reducing the spread of the disease in the general population)
- ❑ It was not recommended by the government
- ❑ It was not recommended by my doctor
- ❑ It is not easy to get a vaccination appointment
- ❑ I heard about a friend/ family who had developed severe side effects from the influenza vaccine
- ❑ Even if I was sick with influenza, I do not think I would infect others with influenza
- ❑ My friends and family discouraged me to take the vaccine
- ❑ I have been previously advised against receiving a vaccination
- ❑ I am afraid of needles
- ❑ **Others: ___**

Q21. Assuming there is a vaccine developed for dengue prevention that is approved by global and local health authorities, how much do you agree with the statements below? Please rate each statement on a scale from 0 to 10, with 0 being 'strongly disagree' and 10 being 'strongly agree'.

		(0)	(1)	(2)	(3)	(4)	(5)	(6)	(7)	(8)	(9)	(10)	
a) I am concerned about the level of protection the vaccine will give me	Strongly disagree												Strongly agree
b) I am concerned that I may need repeated booster vaccines to maintain immunity	Strongly disagree												Strongly agree
c) I am concerned about the vaccine safety and adverse effects	Strongly disagree												Strongly agree
d) I think vaccines are harmful	Strongly disagree												Strongly agree
e) I am concerned that the vaccine will transmit the dengue virus to me	Strongly disagree												Strongly agree
f) I do not want to be the first to try a new dengue vaccine	Strongly disagree												Strongly agree

		(0)	(1)	(2)	(3)	(4)	(5)	(6)	(7)	(8)	(9)	(10)	
g) I will wait to be reassured that there are no safety risks	Strongly disagree												Strongly agree
h) I am concerned that the vaccine may contain heavy metals or dangerous substances	Strongly disagree												Strongly agree
i) I am concerned that the vaccine may cause autism	Strongly disagree												Strongly agree
j) My immune system is weak and I believe that taking the vaccine will cause me more harm	Strongly disagree												Strongly agree
k) I will consider it only for my children or parents	Strongly disagree												Strongly agree
l) I am afraid of needles	Strongly disagree												Strongly agree
m) I don't believe in vaccines	Strongly disagree												Strongly agree

Q22. How much do you agree with the statements below regarding a hypothetical dengue vaccine? Please rate each statement on a scale from 0 to 10, with 0 being 'strongly disagree' and 10 being 'strongly agree'.

		(0)	(1)	(2)	(3)	(4)	(5)	(6)	(7)	(8)	(9)	(10)	
a) I believe that the vaccine should be made accessible to the public including myself	Strongly disagree												Strongly agree
b) I trust the healthcare system and professionals in my country to deliver the vaccine and manage its side effects	Strongly disagree												Strongly agree
c) If I have to pay for the vaccination, I will not do it	Strongly disagree												Strongly agree
d) I will be more willing to get vaccinated if there are incentives (cash, points, or a gift)	Strongly disagree												Strongly agree
e) It depends on how severe the dengue	Strongly disagree												Strongly agree

			(0)	(1)	(2)	(3)	(4)	(5)	(6)	(7)	(8)	(9)	(10)	
	epidemic is/ how common dengue is when I am offered the vaccine													
f)	If the risk of contracting dengue is low, I may not get the dengue vaccine	Strongly disagree												Strongly agree
g)	I think dengue vaccination is more important than other optional vaccines (e.g. influenza)	Strongly disagree												Strongly agree
h)	I am not convinced that it will be effective, look at the influenza/COVID-19 vaccine	Strongly disagree												Strongly agree

Q23. Assuming there is a vaccine developed for dengue prevention that is approved by global and local health authorities, how willing are you to consider getting vaccinated or recommend vaccination against dengue?
Please rate each statement on a scale from 0 to 10, with 0 being 'not willing at all' and 10 being 'very willing'.

			(0)	(1)	(2)	(3)	(4)	(5)	(6)	(7)	(8)	(9)	(10)	
a)	Myself	Not willing at all												Very willing
b)	My spouse (if applicable)	Not willing at all												Very willing
c)	My children (if applicable)	Not willing at all												Very willing
d)	My parents (if applicable)	Not willing at all												Very willing
e)	My friends	Not willing at all												Very willing

Q24. Are you willing to consider a dengue vaccine if it is recommended by your physician?
Please rate this statement on a scale from 0 to 10, with 0 being 'strongly disagree' and 10 being 'strongly agree'.

	(0)	(1)	(2)	(3)	(4)	(5)	(6)	(7)	(8)	(9)	(10)	
Strongly disagree												Strongly agree

DENGUE VACCINATION + VECTOR CONTROL

Q25. Do you think that a dengue vaccination program should be rolled out with other programs, or as a standalone program? *Other programs may include dengue vaccine education, vector control (any method to limit or eliminate mosquitoes, which transmit dengue), vector control education etc.*

- ❍ I believe that a vaccination program should be rolled out with an education program
- ❍ I believe that a vaccination program should be rolled out with a vector control program (any method to limit or eradicate mosquitoes which transmit dengue)
- ❍ I believe that a vaccination program should be rolled out with both an education program and vector control program
- ❍ I believe that a vector control program alone is sufficient
- ❍ I believe that a vaccination program should be a standalone program
- ❍ I don't believe a vaccination program is necessary

Q26. Please share any other reason why you would be willing to get a dengue vaccine.

Q27. Please share any other reason why you would not be willing to get a dengue vaccine.

Q28. Please select which dengue prevention activities the government should allocate more resources to (e.g., funding, subsidizing, distribution, manpower) in the future.

- ❑ Use wire mesh screens, mosquito nets or mosquito coils
- ❑ Drain water from pots and cover all water containers
- ❑ Community mosquito fogging
- ❑ Spraying mosquito repellent and using larvicide to kill mosquito larvae
- ❑ Wolbachia program (WIAM): Natural bacteria called Wolbachia reduce the abilities of Aedes mosquitoes to transmit dengue. Mosquitoes bred with the Wolbachia bacteria are released into nature to spread the bacteria among the wider mosquito population, thus naturally reducing the transmission of dengue.
- ❑ Dengue vaccination
- ❑ Others: ___
- ❑ None of the above

EDUCATION

Q29. Do you agree with the statements below on the impact of religion on your daily decision-making process?
Please rate each statement on a scale from 0 to 10, with 0 being 'strongly disagree' and 10 being 'strongly agree'.

Religion and spirituality may impact decisions related to but not including diet, medicines with animal origins (e.g. halal vaccines, sterile insect treatments, blood transfusions etc.) and preferred gender of doctors or other healthcare professionals

		(0)	(1)	(2)	(3)	(4)	(5)	(6)	(7)	(8)	(9)	(10)	
a) My religious beliefs guide my health decisions	Strongly disagree												Strongly agree
b) I live my life according to my religious beliefs	Strongly disagree												Strongly agree
c) My religion prohibits me from getting vaccinated	Strongly disagree												Strongly agree

Q30. Do you agree with the following statements relating to your community/ government leader(s) (e.g. governors, mayors, councilors etc.)? Please rate each statement on a scale from 0 to 10, with 0 being 'strongly disagree' and 10 being 'strongly agree'. *Community/ government leader(s) can include village chiefs, community health leaders, residential community leaders, etc.*

		(0)	(1)	(2)	(3)	(4)	(5)	(6)	(7)	(8)	(9)	(10)	
a) The opinion of my community/ government leader(s) is important to me	Strongly disagree												Strongly agree
b) My community organizes events promoting health	Strongly disagree												Strongly agree
c) I actively participate in community events, specific to promoting good/improved health and well-being	Strongly disagree												Strongly agree

Q31. Do you agree with the following statements relating to your influencer(s)?
Please rate each statement on a scale from 0 to 10, with 0 being 'strongly disagree' and 10 being 'strongly agree'.
Influences can include social media influencer(s), local and international celebrities etc.

		(0)	(1)	(2)	(3)	(4)	(5)	(6)	(7)	(8)	(9)	(10)	
a) The opinions of my influencer(s) is important to me	Strongly disagree												Strongly agree
b) My influencer(s) organizes events promoting good/	Strongly disagree												Strongly agree

			(0)	(1)	(2)	(3)	(4)	(5)	(6)	(7)	(8)	(9)	(10)	
	improved health and well-being													
c)	I actively participate in health-related events hosted by my influencer(s)	Strongly disagree												Strongly agree

Q32. Which of the following channels do you most commonly use when actively looking for health-related information including information about vaccines? Please select all that apply.

- ❑ Search engines (e.g., Google, Yahoo!)
- ❑ Social Media (e.g., Facebook, Twitter, TikTok, Instagram, YouTube)
- ❑ Messaging platforms (e.g. WhatsApp, Telegram, Facebook Messenger)
- ❑ Consumer reviews
- ❑ Product and brand websites
- ❑ Product and brand blogs/video blogs
- ❑ Websites specializing in health-related information (e.g., WebMD, MayoClinic, Hello Health)
- ❑ Government or health agency websites or portals (e.g. Ministry of Health, Ministerio de Salud, Ministério da Saúde, Kementerian Kesihatan)
- ❑ Patient advocacy websites
- ❑ Scientific journals (e.g. PubMed, New England Journal of Medicine)
- ❑ Online forums, 'Question & Answer' services (e.g., Quora, Reddit)
- ❑ Newspapers, magazines
- ❑ Television
- ❑ Radio
- ❑ Others: ___

Q33. Out of the following channels that you use, please select the top 3 channels that you most commonly use when looking for health-related information.

Q34. Which of the following types of people/organizations do you trust to receive health-related information from?

- ❑ Doctors
- ❑ Nurses/ Paramedics
- ❑ Pharmacists
- ❑ Government
- ❑ Religious leaders
- ❑ Community leaders (e.g. village chiefs, community health leaders, residential community leaders, etc.)
- ❑ Pharmaceutical companies
- ❑ Non-profit organizations (e.g. World Health Organization)
- ❑ Scientific organizations
- ❑ Family, friends and/ or colleagues

- ❑ Influencers (e.g. local and international celebrities, etc.)
- ❑ Others: ___

Q35. Out of the types of people/organizations that you trust, please select the top 3 sources that you trust the most when looking for health-related information.
